# Crebanine Induces Cell Death and Alters the Mitotic Process in Renal Cell Carcinoma In Vitro

**DOI:** 10.3390/ijms26146896

**Published:** 2025-07-18

**Authors:** Hung-Jen Shih, Hsuan-Chih Hsu, Chien-Te Liu, Ya-Chuan Chang, Chia-Ying Yu, Wen-Wei Sung

**Affiliations:** 1 Division of Urology, Department of Surgery, Changhua Christian Hospital, Changhua 500, Taiwan; jasta1206@gmail.com; 2 Department of Post-Baccalaureate Medicine, College of Medicine, National Chung Hsing University, Taichung 402, Taiwan; 3 Department of Urology, School of Medicine, College of Medicine, Taipei Medical University, Taipei 110, Taiwan; 4 School of Medicine, Chung Shan Medical University, Taichung 402, Taiwan; s1001141@gm.csmu.edu.tw (H.-C.H.); kevinliu.5899@gmail.com (C.-T.L.); raptor7037@gmail.com (Y.-C.C.); cyyu2015@gmail.com (C.-Y.Y.); 5 Department of Urology, Chung Shan Medical University Hospital, Taichung 402, Taiwan; 6 Institute of Medicine, Chung Shan Medical University, Taichung 402, Taiwan

**Keywords:** kidney cancer, aporphine alkaloid, *Stephania venosa*, natural product, systemic treatment

## Abstract

Advanced renal cell carcinoma (RCC) has a poor prognosis; this drives the exploration of alternative systemic therapies to identify more effective treatment options. Recent research has revealed that crebanine, an alkaloid derivative of the Stephania genus, induces apoptotic effects in various cancers; however, a thorough investigation of the role of crebanine in RCC has not been conducted thus far. For this study, we evaluated tumor cell viability, clonogenicity, cell-cycle distributions, morphological changes, and cell mortality with the aim of exploring the antitumor effects of crebanine in RCC. Furthermore, we compared gene and protein expressions using RNA sequencing analysis and Western blotting. The findings indicated that crebanine significantly inhibited RCC colonies and caused G1-phase cell-cycle arrest with sub-G1-phase accumulation, thus leading to suppressed cell proliferation and cell death. In addition, Hoechst 33342 staining was used to observe apoptotic cells, which revealed chromatin condensation and a reduction in the nuclear volume associated with apoptosis. Further, gene ontology (GO) and Kyoto Encyclopedia of Genes and Genomes (KEGG) analysis indicated that differentially expressed genes are involved in the initiation of DNA replication, centrosome duplication, chromosome congression, and mitotic processes in the cell cycle along with signaling pathways, such as I-kappaB kinase/NF-kappaB signaling, Hippo signaling, and intrinsic apoptotic pathways. Consistent with GO and KEGG analyses, increased levels of cleaved caspase-3, cleaved caspase-7, and cleaved PARP, and decreased levels of cIAP1, BCL2, survivin, and claspin were observed. Finally, the expressions of G1/S phase transition cyclin D1, cyclin E/CDK2, and cyclin A2/CDK2 complexes were downregulated. Overall, these findings supported the potential of crebanine as an adjuvant therapy in RCC.

## 1. Introduction

According to GLOBOCAN data, renal cell carcinoma (RCC) is the fourteenth most frequently diagnosed and the sixteenth most lethal cancer of all sites, with 434,419 diagnoses and 155,702 deaths globally in 2022 [1]. Across the globe, age-standardized incidence rates and mortality rates (per 100,000 persons/year) were 5.9 and 2.0 for men and 3.0 and 0.9 for women, respectively [1]. In [2], among all diagnoses in a study, clear-cell RCC, papillary RCC, and chromophobe RCC accounted for 75%, 10%, and 5%, respectively. Although early stages of RCC appeared asymptomatic in [3,4], 20–30% of patients presented with metastasis at initial diagnosis. A few studies have indicated that while cytoreductive nephrectomy is the first-line systemic treatment option for metastatic RCC (mRCC) patients, immune checkpoint inhibitors and adjuvant target therapies are the options for others [5,6]. According to the Surveillance, Epidemiology, and End Results (SEER) database, between 2013 and 2019, the five-year overall survival rates of unstaged and distant-stage RCC were 53.25% and 17.4%, respectively [7]. Despite the disease control rate of immune checkpoint inhibitors and adjuvant target therapy being susceptible to IMDC scores, only one-third of the objective response rates were observed in mRCC patients, regardless of the therapy line in real-world practice [8,9]. However, a meta-analysis revealed that first-line systematic therapy prolonged patients’ lives for an average of 28–41 months, thus leading to an average of 40–61% risk of serious side effects, depending on treatment cocktails [10]. Clearly, patients diagnosed with advanced RCC required more second- or third-line treatment options, which led to an urgent need for adjunct therapy.

Crebanine is an isoquinoline-like alkaloid isolated from plants of the *Stephania* genus [11]. In another study, crebanine induced ROS-dependent apoptosis in human hepatocellular carcinoma through the PI3K/AKT pathway, which decreased superoxide, glutathione peroxidase, and malondialdehyde [12]. In ovarian cancer, crebanine showed a chemosensitizing effect by inducing apoptotic cell death and inhibiting cisplatin-induced resistant pathways [13]. In human lung adenocarcinoma, crebanine reduced NF-κB pathway activation that blocks apoptosis [14]. A study revealed that crebanine promoted cleaved caspase-3, -8, -9, and cleaved PARP in a matter of time, which sensitized a reduction in mitochondrial membrane potential that leads to apoptosis in leukemia and cervical cancer cells [15]. Although the benefits of crebanine in various cancer treatments have been discovered, the underlying mechanism of crebanine regulation in RCC remains unknown.

The poor prognosis in advanced RCC drives the search for systemic therapy agents. Crebanine has proven to be a therapeutic agent in many types of cancer; however, the role of crebanine as a potentially effective anticancer agent in RCC has not yet been confirmed. Thus, the main purpose of this study was to examine the anticancer effects of crebanine in the 786-0, A498, and Caki-1 cell lines and elucidate the effectiveness of crebanine in cell-cycle phase distribution and apoptosis. With the aid of a next-generation sequencing technique, the researchers of this study hope to explore its functional and biological genes. The results of this study elucidate the antitumor role of crebanine in RCC and, hopefully, provide an adjuvant agent for clinical use.

## 2. Results

### 2.1. Crebanine Prohibits Cell Proliferation and Promotes Apoptosis in 786-0, A498, and Caki-1 Cells In Vitro

**Figure 1:**
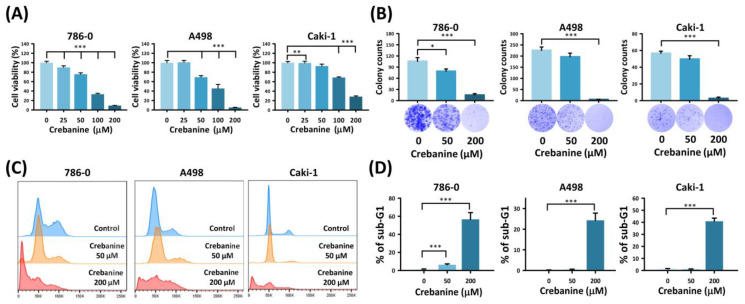
Crebanine inhibits RCC cell growth in a potent apoptotic manner. (**A**) Crebanine reduced the cell viability of the 786-0, A498, and Caki-1 cells, as determined by the MTT assay, where 25 µM, 50 µM, 100 µM, and 200 µM of crebanine and DMSO were used in the MTT assay, respectively. (**B**) While evaluating the growth changes in 786-0, A498, and Caki-1 cells in 50 µM and 200 µM crebanine, the latter was found to inhibit the formers’ colony formations. (**C**,**D**) Crebanine increased RCC cells arrested in the sub-G1 phase. Cell-cycle distributions were evaluated by flow cytometry. Cell-cycle distributions were shown for the 786-0, A498, and Caki-1 cells. Data were shown as the mean ± SD (* *p* < 0.05, ** *p* < 0.01; *** *p* < 0.001).

Cell viability tests were used to elucidate the therapeutic potency of crebanine in the 786-0, A498, and Caki-1 cell lines. The decreased cell viability with dose response suggested crebanine’s cytotoxic effect. Based on MTT assay results, the IC_50_ values of crebanine for 786-0, A498, and Caki-1 cells were approximately 77.4 µM, 108.6 µM, and 130.5 µM, respectively. Therefore, we selected concentrations ranging from 50 µM–200 µM to cover sub- and supra-IC_50_ conditions for further evaluation of biological responses (Figure 1A). As depicted in Figure 1B, the number of colonies of 786-0, A498, and Caki-1 significantly decreased when treated with 200 µM crebanine (15.0 ± 3.6%, 5.0 ± 1%, and 2.7 ± 1.5%, respectively). Furthermore, a significant rise in 786-0, A498, and Caki-1 arrested in the sub-G1 phase was observed (56.6 ± 7.46%, 24.3 ± 3.49%, and 40.87 ± 2.62%, respectively; Figure 1C,D). This evidence revealed that crebanine has the potential to inhibit cell viability and induce cell death in RCC cells.

**Figure 2:**
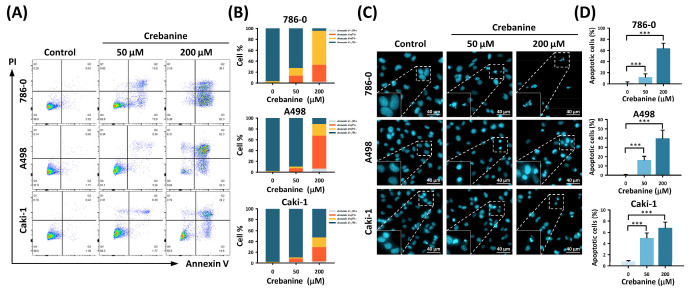
Crebanine promotes apoptosis in RCC cell lines. (**A**) Annexin V/PI staining was performed with flow cytometry in 786-0, A498, and Caki-1 cells, which were treated with crebanine (0 µM, 50 µM, and 200 µM) for 48 h. (**B**) The proportions of Annexin^+/−^ PI^+/−^ distribution of 786-0, A498, and Caki-1 cells were treated with crebanine (0 µM, 50 µM, 200 µM) for 48 h, which are depicted as bar charts. (**C**) Apoptotic cells with morphological changes in nuclei increased in response to crebanine treatment. 786-0, A498, and Caki-1 cells were treated with crebanine (0, 50, and 200 µM) for 24 h. (**D**) The quantity of apoptotic cells in response to treatment is depicted in percentage units. Data are presented as the mean ± SD (*** *p* < 0.001).

In addition, flow cytometry with Annexin V/PI dual staining revealed early and late apoptotic cell patterns after treatment with crebanine for 48 h (93.1 ± 1.21%, 82.5 ± 1.85%, and 44.13 ± 5.16%, respectively; Figure 2A,B). Cells treated with 50 µM and 200 µM crebanine for 24 h showed accumulation of apoptotic cells with Hoechst stain 33342. Morphological changes, including chromatin condensation and nuclear volume reduction, accompanied apoptosis in the treated RCC cells. When treated with 200 µM crebanine, apoptotic RCC cells increased from 1.78 ± 1.72% to 63.8 ± 9.13% in 786-0, from 0.41 ± 0.56% to 39.77 ± 8.84% in A498, and from 0.82 ± 0.13% to 6.83 ± 0.99% in Caki-1 compared to the control groups (all *p* < 0.001, Figure 2C,D). Fluorescence microscopy complying with Annexin V/PI dual staining flow cytometry indicated that crebanine potentially promoted cellular apoptosis in RCC cells.

### 2.2. Crebanine Alters the Mitotic Process and the IκB Kinase/NF-κB Signaling Pathway in Differential Gene Analysis

**Figure 3:**
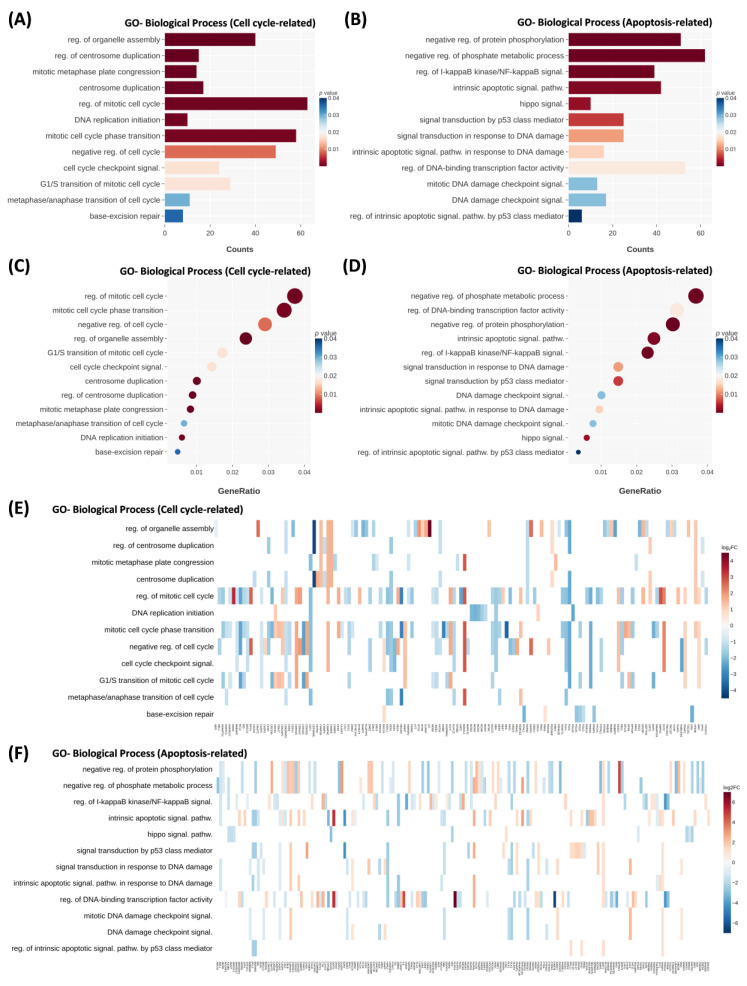
Differential gene expression analysis of renal cell carcinoma. (**A**,**B**) Cell-cycle-related and apoptosis-related GO biological process bar chart. The *p*-value is depicted in color. (**C**,**D**) Cell-cycle-related and apoptosis-related GO biological processes are presented by the dot chart. The *p*-value is depicted in color. The size of the dots reflects the gene ratio. (**E**,**F**) Cell-cycle-related and apoptosis-related GO biological processes are depicted by the heat map. Log2-fold change is depicted in color.

The crebanine model was further compared to the control group with DNA sequencing data. The RNA-seq data analysis is presented in Supplementary Table S2. Alterations in biological process genes revealed an upregulated or downregulated cell cycle and apoptosis pathways. Cell-cycle pathways—which include the regulation of organelle assembly, regulation of centrosome duplication, mitotic metaphase plate congression, centrosome duplication, regulation of mitotic cell cycle, DNA replication initiation, and mitotic cell-cycle phase transition—were the most closely associated (Figure 3A,C). The negative regulation of protein phosphorylation, negative regulation of the phosphate metabolic process, regulation of I-kappaB kinase/NF-kappaB signaling, intrinsic apoptotic signaling pathway, and Hippo signaling were the most closely associated pathways (Figure 3B,D). Notably, the mitotic arrest deficient 1 like 1 (MAD1L1) gene—a spindle assembly checkpoint—was upregulated; the CHMP4C gene, which belongs to the chromatin modifying protein/charged multivesicular body protein family, was upregulated (Figure 3E,F).

**Figure 4:**
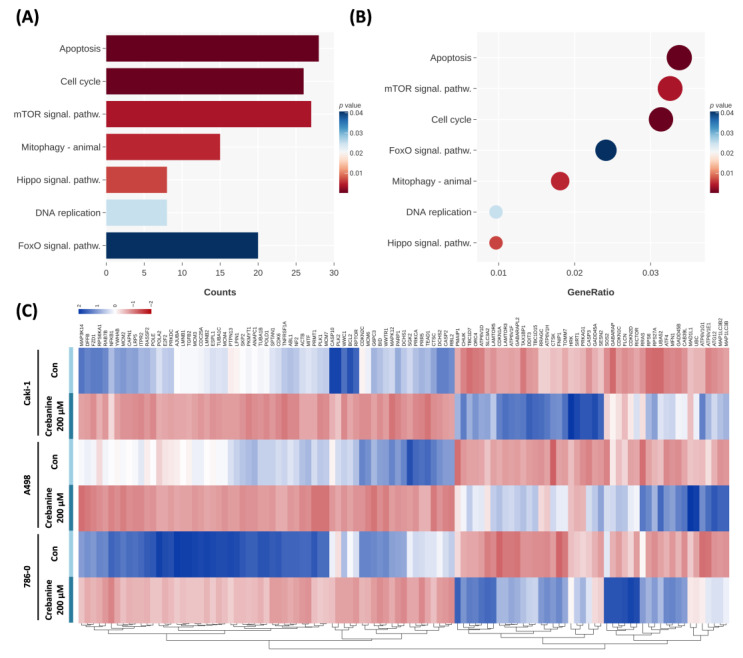
Differential gene expression analysis of renal cell carcinoma. (**A**,**B**) KEGG analysis of the models. (**C**) Gene heat map of the model. Downregulated genes are depicted in red. Upregulated genes are depicted in blue.

The KEGG analysis results were consistent with the GO analysis. The apoptotic and cell-cycle pathways were regulated (Figure 4). Moreover, the mTOR signaling, mitophagy-animal, and Hippo signaling pathways were found to be associated with crebanine gene regulation. The observed changes in Caki-1, A498, and 786-0 RCC cells were consistent.

### 2.3. Crebanine Exerts Its Anticancer Effects Through the Promotion of Apoptosis and Regulation of the G1/S Phase Transition

**Figure 5:**
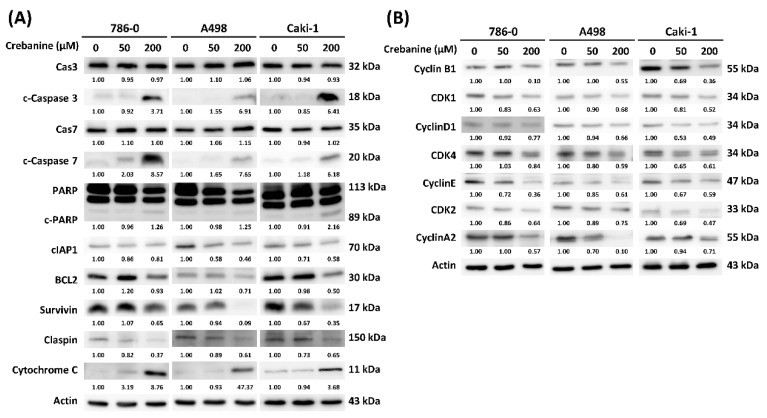
Validation of apoptosis and cell-cycle-related proteins via Western blotting. The protein expression changed within 24 h. The 50 µM or 200 µM crebanine treatments on RCC cells were measured by Western blotting, including (**A**) apoptosis-related proteins and (**B**) cell-cycle-related proteins.

To explore the underlying mechanisms altered by crebanine in RCC cells, Western blotting was performed. The blot images are presented in Figure 5A, while the quantitative results are displayed as bar charts in Supplementary Figure S2. RCC cells were treated with 50 µM and 200 µM crebanine; the protein expressions of cleaved caspase-3 and cleaved caspase-7, which were the executioners of intrinsic or extrinsic apoptotic pathways, cleaved the downstream substrates. Further, high doses of crebanine reversed the conversion of PARP to cleaved PARP. This led to the downregulation of PARP in 786-0 and A498 cells; slight downregulation of PARP in Caki-1 cells; and the upregulation of cleaved PARP protein in 786-0, A498, and Caki-1 cells. The antiapoptotic proteins—including clAP1, BCL2, survivin, and claspin—were downregulated by crebanine. Finally, cytochrome c protein was present in a high dose of crebanine.

In terms of cell-cycle proteins, cyclin B1/CDK1 protein complexes were downregulated in the presence of crebanine. The G1–S cell-cycle regulator cyclin D1 was downregulated in RCC cells. Moreover, the loss of CDK4/cyclin E was noted when doses of crebanine were higher. The depletion of CDK2/cyclin A2 was observed in 200 µM crebanine (Figure 5B).

## 3. Discussion

The mRCC was characterized by a low survival rate due to a high possibility of resistance to medication, even when it was initially effective. Overall, the present study demonstrated that crebanine inhibited tumor growth through the cell cycle and apoptosis in RCC cell lines. Our focus was the entire genome level and consequential changes to gene products. The present study demonstrated that crebanine regulates DNA replication initiation, centrosome duplication, chromosome congression, and mitotic processes in the cell cycle and I-kappaB kinase/NF-kappaB, the Hippo, and intrinsic apoptotic signaling pathways in apoptosis. These findings supported the hypothesis regarding the use of crebanine as a potential adjuvant therapy for RCC.

Crebanine is a derivative alkaloid of the *Stephania* genus, which has anticancer activity, as previously reported [11]. Aporphine alkaloids are stable under physiological pH and attain equilibrium in an alkaline environment [16]. Most aporphine alkaloids exhibit low oral absolute bioavailability, with reported values of 4.7% for boldine, 4.6% for nuciferine, and 17.5% for liriodenine in rats. These compounds typically reach peak plasma concentration (T_max_) within three hours, with the maximum concentration (C_max_) exceeding 10 ng/dL in the bloodstream. Moreover, aporphine alkaloids widely distribute to various organs such as lungs, heart, spleen, and kidneys. These compounds are generally eliminated quickly, with a half-life (T_1/2_) of less than three hours and a high clearance rate (over 50 L/kg/h), primarily through metabolism by cytochrome P450 enzymes and uridine diphosphate glycosyltransferases (UGTs) [17]. Rapid absorption, broad tissue distribution, and fast elimination but low oral bioavailability characteristics may limit the systemic efficacy of aporphine alkaloids. As shown in previous research, protoberberine (tetrahydropalmatine and N-methyltetrahydropalmatine) and aporphine alkaloids (crebanine and O-methylbulbocapnine) inhibit leukemic cell growth at lower concentrations and show synergic effects on cisplatin-resistant ovarian cancer cells [13,14]. A few researchers have also evaluated the apoptotic effect of crebanine at 105 µM on human hepatocellular carcinoma and received promising feedback [12]. In another study, the combination of cisplatin and crebanine revealed an increase in apoptotic cells, while crebanine at 60 µM alone did not significantly cause apoptosis in ovarian cancer cells [13]. Previous research has also revealed that 30 µg/mL (88.5 µM) crebanine had little cytotoxic effect on normal fibroblast cells, thus reducing cell viability to 72% and effectively inducing G1-phase cell-cycle arrest in human cancer cells [15]. In our study, cell viability tests were performed on RCC cells at concentrations of 25 µM, 50 µM, 100 µM, and 200 µM. Furthermore, 50 µM and 200 µM of crebanine were used for further analysis, and a higher dosage of crebanine significantly promoted apoptosis. These findings suggest that crebanine can be a safe and effective agent for RCC cells.

The results of the present study suggest that crebanine promotes cell-cycle arrest in the sub-G1 phase (Figure 1). The researchers of this study verified that crebanine inhibited G1/S phase transition cyclin D1, cyclin E/CDK 2, and cyclin A2/CDK 2 complexes via Western blotting (Figure 5). RCC was characterized by mutation in TP53, loss of p27, and increased expression of Ki-67 [18]. Recent studies have discovered frequent mutations of the tumor suppressor protein BAP1 (BRCA1-associated protein 1) and PBRM1 (polybromo 1) in RCC [19,20]. BAP1 has been found to be associated with chromosomal stability, which is maintained by mitotic spindle-regulating microspherule protein 1 (MCRS1) [21]. The loss of PBRM1 was noted to lead to increased HIF transcription, STAT3, and mTOR signaling, thereby favoring tumor growth [21]. Furthermore, cyclin D1 led to retinoplastoma protein (pRB) phosphorylation and drove RCC from the G1 phase to the S phase, thereby accelerating tumor cell proliferation [22]. In short, genetic mutations that cause dysregulation in G1/S phase transition are a few of the major causes of cell proliferation in RCC. In this research, differential gene analysis consistent with Western blotting confirmed that crebanine inhibits cell proliferation and induces G1/S phase cell-cycle arrest.

Further, the intrinsic apoptosis pathway was initiated by caspase-2, caspase-8, caspase-9, and caspase-10, and executed by caspase-3, caspase-6, and caspase-7 [23]. Subsequently, BIM, BID, and PUMA activated the proapoptotic sensitizer protein Bax and Bak, thereby disrupting mitochondrial outer membrane permeability (MOMP) [24]. CDKs and p53 regulated apoptosis by mediating antiapoptotic proteins, including BCL-2, BCL-X, and MCL1 [25]. The observation of differential responses in RCC cells in Annexin V/PI and Hochest stain 33342 may have been caused by a unique gene mutation in RCC cell lines. According to the Cancer Cell Line Encyclopedia (CCLE) database, there are 786-0 mutates in the PTEN and TP53 (p.R248W) genes, A498 mutates in the SETD2 (p.V2536fs) and MLL3 (p.G2986D) genes, and Caki-1 mutates in SETD2 and MET (p.V1238I), [26]. In the present study, crebanine was found to induce apoptosis, as indicated by flow cytometry and Hoechst 33342 staining. Apoptotic-related genes regulated by crebanine have been extensively studied, and apoptosis has been associated with a negative control of tumor cell growth—for example, protein phosphorylation. Moreover, Yodkeeree et al. reported a similar apoptotic effect of crebanine in downregulating I-kappaB kinase/NF-kappaB signaling [14]. In addition, activated cleaved caspase-3 and caspase-7—which execute apoptosis with downregulated antiapoptotic proteins clAP1, BCL2, survivin, and claspin—were also detected. PARP-1 was found to play an important role in apoptosis, which is frequently dysregulated in tumor cells [27]. Olaparib and talazoparib have been widely used in other tumor cells, while the inhibition of PARP has been found to act as a promising approach for RCC therapy [28,29]. Therefore, crebanine was found to induce apoptosis potentially through the activation of apoptosis executioners, inhibition of antiapoptotic protein, and conversion of PARP to cleaved PARP.

Further, crebanine-induced G1/S phase arrest may play a mechanistic role in triggering apoptosis in RCC cells. Previous studies have revealed that DNA damage or oncogenic stress can activate p53-dependent checkpoints, thus leading to p21-mediated cell-cycle arrest and the induction of pro-apoptotic proteins, such as BAX and PUMA [23,30]. Conversely, apoptosis can also feedback into the cell-cycle machinery by activating caspases, which degrade cyclins and CDKs, thereby amplifying cell death [31]. Therefore, the observed G1/S arrest is likely a primary event that contributes to apoptosis, although reciprocal interactions may also exist.

Tumorigenesis and tumor persistence are key mechanisms underlying RCC pathogenesis. Von Hippel–Lindau protein (pVHL) and hypoxia-inducible factor (HIF) signaling pathways play a crucial role in pathogenesis in RCC [32]. Commonly observed in both sporadic and hereditary RCC, loss-of-function mutations in the VHL gene disrupt normal oxygen-sensing mechanisms by preventing the degradation of HIF2α [33]. Notably, HIF2α functions as an oncogene in ccRCC, whereas HIF1α may play a tumor-suppressive role, thereby illustrating the complexity of HIF signaling in this cancer type [32]. Although our current study did not directly assess VHL or HIF pathway activity, this signaling axis remains a critical therapeutic target in ccRCC, as evidenced by the clinical success of HIF2α inhibitors such as belzutifan. Future transcriptomic or proteomic analyses of crebanine-treated RCC could further explore whether the pVHL–HIF signaling pathway is modulated as part of crebanine’s antitumor mechanism. Cancer stem cell (CSC) biology also emphasizes the importance of therapeutic resistance. CSCs exhibit a remarkable capacity to switch between glycolysis and oxidative phosphorylation, and frequently rely on fatty acid metabolism to meet their energy demands in nutrient-limited or hypoxic tumor microenvironments. This metabolic plasticity not only supports the survival and self-renewal of CSCs but also contributes to their capacity to add resistance under metabolic stress [34].

A significant number of patients have been found to develop grade-3 or higher adverse events when treated with current RCC therapies; moreover, diarrhea, cardiovascular events, decreased appetite, fatigue, and nausea are among the most common problems. Sunitinib is the first-line treatment for advanced RCC, although 77–92% of patients receiving vascular endothelial growth factor pathway inhibitors experience adverse cardiovascular events of any grade or proteinuria during the treatment period [35]. Moreover, patients may acquire resistance to sunitinib within 6–15 months of therapy through mechanisms that involve proangiogenic pathways, the tumor microenvironment, and the endoplasmic reticulum stress response [36]. Pembrolizumab was found to improve overall and disease-free survival in patients with intermediate- and high-risk clear-cell RCC compared to the placebo in the KEYNOTE-564 trial [37,38]. A network meta-analysis involving 17 studies and 14,298 patients demonstrated that pembrolizumab significantly increased disease-free survival than a placebo [39]. In the CLEAR trial, the combination of lenvatinib and pembrolizumab showed superiority over sunitinib in terms of overall survival and progression-free survival; however, 82–84% of patients experienced grade-3 or higher adverse events [40,41]. In the Phase III CheckMate 914 trial, nivolumab monotherapy did not improve disease-free survival, and nivolumab plus ipilimumab resulted in higher rates of grade-3 or greater adverse events than the other groups, as observed in both the trial and in previous meta-analyses [39,42].

Further, positive surgical margins are an independent predictor of worse overall survival in patients with RCC, particularly in AJCC stages II–IV. Positive surgical margins were associated with a 43% increased risk of death and were particularly prognostic in higher-stage disease. Notably, among patients who would otherwise meet the criteria for adjuvant therapy (e.g., KEYNOTE-564), positive surgical margins were linked to a 62% higher mortality risk, thereby suggesting future risk stratification models and consideration for adjuvant treatment eligibility [43]. The present study aimed to explore the use of crebanine against RCC, as previous findings indicate that crebanine displays selective cytotoxicity toward cancer cell lines while sparing normal human fibroblasts [14,15,44].

The recent guidelines for treating RCC include antiangiogenic drugs, immune checkpoint inhibitors, mTOR inhibitors, and other immunotherapies or cytokines; however, they do not currently include agents that specifically regulate the NF-κB or Hippo signaling pathways [45]. Numerous compounds targeting the NF-κB pathway have been discovered, such as nonsteroidal anti-inflammatory drugs (NSAIDs), dexamethasone, thalidomide, monoclonal antibodies, proteasome inhibitors, and various natural agents [46]. However, the clinical use of these agents in cancer therapy has been limited due to their side effects (e.g., immunosuppression) or low bioavailability, thus prompting investigations into combining them with immunotherapy [47,48,49]. For example, curcumin—an inhibitor of IκB kinase—promotes tumor infiltration of antitumor T cells and PD-1 ubiquitination when combined with programmed cell death protein 1 (PD-1) blockade [50]. Moreover, dual targeting of cytotoxic T-lymphocyte-associated protein 4 (CTLA-4) and PD-1, alongside clinically available TNF inhibitors, enhances macrophage depletion and prevents colitis and hepatitis in vivo [51]. In addition, anti-PD-1 therapy—combined with a CDK4/6 inhibitor—suppresses NF-κB activity and PD-L1 expression [52].

Several agents that regulate the Hippo signaling pathway have entered clinical trials. Transcriptional enhanced associate domain (TEAD) family inhibitors, such as VT3989 and IK-930, are being evaluated for treating solid tumors and mesothelioma, while yes-associated protein (YAP) antisense oligonucleotide ION537 is being tested for advanced solid tumors [53,54]. XMU-MP-1, an inhibitor of mammalian sterile 20-like protein kinase 1 (MST1) activity, has been shown to reduce the progression of breast cancer in vivo [55]. In conclusion, further in vivo investigations are necessary to clarify the potential therapeutic benefits of combining crebanine with immunotherapy and elucidate its underlying mechanisms.

Previous in vivo studies have also provided insight into the potential therapeutic effects of crebanine. Yangqiu et al. established a middle cerebral artery occlusion and reperfusion model in adult Sprague Dawley rats, using crebanine at doses of 250 mg/kg and 500 mg/kg [56]. Crebanine was found to attenuate surgery-induced degenerative changes, thereby inhibiting oxidative stress and suppressing neuroinflammation in vivo [56]. In another study, eight-week old C57BL/6J mice were treated using intramuscular injections of crebanine (5 mg/kg) with methy-prednisolone (20 mg/kg) to compare the protective effect of crebanine in femoral head necrosis with other groups [57]. Shankun et al. reported significant restoration of bone volume and trabecular thickness [57]. These in vivo studies suggest the anti-inflammatory and tissue-protective properties of crebanine across different in vivo disease models, thereby supporting its pharmacological application.

It must be noted that this study is limited to in vitro experiments and lacks in vivo validation. First, this study was limited to three RCC cell lines and, thus, may fail to represent the heterogeneity of all RCC subtypes. Second, in vitro results may not accurately reflect crebanine in in vivo studies; thus, the safe and effective concentration of crebanine in animal models or human RCC remains to be determined. Third, the dosage in this study is relatively high in comparison to that utilized in previous studies [12,58], thereby highlighting the need to assess crebanine’s potential cytotoxicity at high dosages in normal cells. We hope our in vitro study of crebanine paves the way for future animal studies and the development of promising and safe treatment options.

## 4. Materials and Methods

### 4.1. Cell Culture

For this study, three RCC cell lines—786-0, A498, and Caki-1—were purchased from the American Type Culture Collection (ATCC, Manassas, VA, USA) and stored according to the supplier’s instructions. The 786-0 and Caki-1 cell lines were maintained in RPMI-1640 medium, while the A498 cell line was maintained in Dulbecco’s Modified Eagle Medium. The media were supplemented with 10% fetal bovine serum, 100 µg/mL streptomycin, 100 U/mL penicillin, 2 g/mL NaHCO_3_ (Thermo Fisher Scientific Inc., Waltham, MA, USA), 1 mM sodium pyruvate, and 0.1 mM nonessential amino acids. All cell lines were cultured in an incubator with 37 °C temperature and 5% CO_2_.

### 4.2. The MTT Assay

The MTT assay was utilized to assess the cytotoxicity of drugs and their impacts on cell growth. Initially, 10^4^ cells were seeded into a 96-well plate and allowed to adhere overnight. Various concentrations of crebanine (MedChemExpress, Monmouth Junction, NJ, USA) were added every other day, followed by a 24 h incubation. After removing the cell supernatant, 100 µL MTT solution (0.5 mg/mL) was added to each well and incubated for 3 h. Subsequently, the cell supernatant was removed and DMSO was added to dissolve the purple formazan product, thus terminating the reaction. Finally, an ELISA reader was used to measure the absorbance at 570 nm. The experiment was performed in triplicate, and the cell viability of each group was calculated and compared to that of the control group.

### 4.3. The Colony Formation Assay

In a 6-well plate, 250–500 RCC cells were seeded and incubated overnight to allow for cell attachment. After treating the cells with crebanine for 24 h, the drug-containing culture medium was removed and drug-free medium was added. The cells were then cultured for another nine days to allow the colonies to grow. On the tenth day, the cells were washed twice with PBS, fixed with ice-cold 95% ethanol, and stained with 0.5% crystal violet. Colony counts were calculated, and each experiment was performed in triplicate.

### 4.4. Cell Cycle and Apoptosis Analysis

In the experiment, flow cytometry (FACSCanto™ II Cell Analyzer; BD Biosciences, Franklin Lakes, NJ, USA) was utilized to analyze the cell-cycle distribution and apoptosis percentage in each group. Cells were seeded in 6-well plates overnight and treated with 0 µM, 50 µM, and 200 µM crebanine for 48 h, after which the cells and their supernatants were collected. For cell-cycle distribution analysis, the cells were fixed in 70% (*v*/*v*) cold ethanol. The fixed cells were then resuspended in PBS containing 0.4 µg/mL PI and 0.5 mg/mL RNase. Furthermore, the cell-cycle phase distribution was assessed using flow cytometry. For the cell apoptosis test, the Annexin V-FITC Apoptosis Detection Kit (Strong Biotech Corporation, Taipei, Taiwan) was used following the manufacturer’s instructions. The cells were resuspended in 100 µL of binding buffer, and 2 µL each of Annexin V-FITC and PI were added. The mixture was incubated in the dark for 15 min. Finally, the percentage of cell apoptosis was measured using flow cytometry. The experiment was performed in triplicate, and the percentages of DNA content in different phases of the cell cycle and cell apoptosis were analyzed using flowjo software (version 10.10, bd Biosciences, Franklin Lakes, NJ, USA).

### 4.5. Nuclear Morphology Analysis Using Hoechst Staining

Hoechst 33342 nuclear staining was used to detect cell apoptosis through morphological changes. RCC cell lines were seeded on a 6-well plate at a density of 1.5 × 10^4^ cells and incubated overnight. The cells were then treated with crebanine at concentrations of 0 µM, 50 µM, and 200 µM for 24 h. After treatment, the cells were washed with PBS, and then 10 µg/mL Hoechst 33342 (Invitrogen, Waltham, MA, USA) was added. The cells were incubated with the stain for 20 min, and their images were captured using an ImageXpress PICO fluorescence microscope (Version 2, San Jose, CA, USA) at 20× magnification. Apoptotic cells emitting blue fluorescence were identified by their characteristic nuclear morphological changes. To quantify apoptosis, five random fields of view were selected, and the percentage of apoptotic cells in each group was calculated.

### 4.6. RNA Extraction and Next-Generation Sequencing

In this study, three RCC cell lines—A498, 786-0, and Caki-1—were treated with crebanine (0 µM and 200 µM) for 48 h. Total RNA was extracted using Trizol^®^ reagent (Invitrogen, Waltham, MA, USA) according to the manufacturer’s instructions, which was subjected to analysis. The RNA quality was assessed using a BioAnalyser (Agilent Technologies, Santa Clara, CA, USA), with all RIN values above 8.2. Detailed RNA quality and quantity data are presented in Supplementary Table S1, and the RNA electropherograms are included in Supplementary Figure S1. Following standard official protocols, sample preparation, library preparation, sequencing, alignment, and differential expression analysis were performed using Genomics (Taipei, Taiwan). Genes with a *p* ≤ 0.05 and a log2-fold change ≥ 2 were considered to be significantly differentially expressed. In addition, representative gene ontology (GO) terms and enriched KEGG pathways were analyzed using the comparative cluster function of the R package clusterProfiler (version 4.7.1). The top 10 enriched GO terms and KEGG pathways were visualized with a cutoff criterion of *p* < 0.05. Finally, the most enriched pathway-related genes were identified for subsequent analysis.

### 4.7. Western Blotting

The cells treated with crebanine for 24 h were lysed using RIPA buffer containing a protease inhibitor cocktail (Roche Molecular Biochemicals, Basel, Switzerland) to extract the proteins. The lysate was then centrifuged at 13,800× *g* for 15 min at 4 °C. The supernatant was collected after discarding the precipitate. Protein concentration was determined using the Bio-Rad Protein Assay (Bio-Rad Laboratories Inc., Hercules, CA, USA), and protein extracts from each group were prepared into electrophoresis samples with equal concentrations. An equal amount of protein from each sample was subjected to SDS-PAGE for electrophoresis. Following electrophoresis, the proteins were transferred onto an Immobilon™-P transfer membrane (Merck Millipore, Burlington, MA, USA). After the transfer, the membrane was blocked with a nonfat milk blocking buffer for 2 h and then incubated with the primary antibody overnight at 4 °C. After 16 h, the membranes were incubated with an HRP-conjugated secondary antibody for 1 h at room temperature. After washing the membranes, they were treated with Immobilon Western Chemiluminescent HRP Substrate (Merck Millipore, Burlington, MA, USA), and luminescence imaging was performed using the ImageQuant LAS4000 analysis system (GE Healthcare, Marlborough, MA, USA).

### 4.8. Statistical Analysis

Statistical analysis was performed using IBM SPSS software (version 20.0, Armonk, NY, USA). Data are presented as the mean ± standard deviation (SD). In addition, Student’s *t*-test was used to analyze discrete or continuous data. All statistical tests were two-sided (SEM), and a *p*-value of less than 0.05 was considered statistically significant (* *p* < 0.05; ** *p* < 0.01; *** *p* < 0.001).

## 5. Conclusions

Crebanine induces cell-cycle arrest in G1/S phase transition and promotes apoptosis. The inhibition of key regulators in G1/S phase transition cyclin D1, cyclin E/CDK 2, and cyclin A2/CDK 2 complexes underlies the mechanisms of the antiproliferation effect. Furthermore, crebanine activates caspase executioners and inhibits antiapoptotic proteins clAP1, BCL2, survivin, and claspin, which induce apoptosis. I-kappaB kinase/NF-kappaB, Hippo, and intrinsic apoptotic signaling pathways might be responses related to the therapeutic mechanism of crebanine. Hence, this study’s findings support the potential efficacy of crebanine as an adjunct therapy for RCC.

## Data Availability

The data that support the findings of this study are available from the corresponding author upon reasonable request.

## Supplementary Materials

The following supporting information can be downloaded at: https://www.mdpi.com/article/10.3390/ijms26146896/s1.

## Abbreviations

AKT	protein kinase B
BAP1	BRCA1-associated protein 1
BCL2	B-cell lymphoma 2
BID	BH3-interacting domain death agonist
BIM	B-cell lymphoma 2-like protein 11
CDK	cyclin-dependent kinases
cIAP1	cellular inhibitor of apoptosis 1
CTLA-4	cytotoxic T-lymphocyte-associated protein 4
DMSO	dimethyl sulfoxide
DNA	deoxyribonucleic acid
ELISA	enzyme-linked immunosorbent assay
G1 phase	gap1 phase
GLOBOCAN	Global Cancer Observatory: Cancer Today
GO	gene ontology
HIF	hypoxia-inducible factor
KEGG	Kyoto Encyclopedia of Genes and Genomes
MCL1	myeloid cell leukemia sequence 1
MCRS1	microspherule protein 1
MET	MET proto-oncogene, receptor tyrosine kinase
MLL3	mixed lineage leukemia 3
MOMP	mitochondrial outer membrane permeability
mRCC	metastatic renal cell carcinoma
MST1	mammalian sterile 20-like protein kinase 1
mTOR	mammalian target of rapamycin
MTT assay	3-(4,5-Dimethylthiazol-2-yl)-2,5-Diphenyltetrazolium Bromide assay
NF-κB	nuclear factor kappa B
NSAIDs	non-steroidal anti-inflammatory drugs
PARP	poly (ADP-ribose) polymerase
PBRM1	polybromo 1
PBS	phosphate-buffered saline
PD-1	programmed cell death protein 1
PD-L1	programmed cell death ligand 1
PI3K	phosphatidylinositol 3-kinase
pRB	retinoblastoma protein
PTEN	phosphatase and tensin homolog
PUMA	p53 upregulated modulator of apoptosis
RCC	renal cell carcinoma
RNA	ribonucleic acid
ROS	reactive oxygen species
SDS-PAGE	sodium dodecyl sulphate-polyacrylamide gel electrophoresis
SEER	Surveillance, Epidemiology, and End Results
SETD2	SET domain containing 2, histone lysine methyltransferase
STAT3	signal transducer and activator of transcription 3
S phase	synthesis phase
TEAD	transcriptional enhanced associated domain
TP53	tumor protein p53
UGTs	uridine diphosphate glycosyltransferases
YAP	yes-associated protein

## References

[1] Bray F., Laversanne M., Sung H., Ferlay J., Siegel R.L., Soerjomataram I., Jemal A. (2024). Global cancer statistics 2022: GLOBOCAN estimates of incidence and mortality worldwide for 36 cancers in 185 countries. CA Cancer J. Clin..

[2] Muglia V.F., Prando A. (2015). Renal cell carcinoma: Histological classification and correlation with imaging findings. Radiol. Bras..

[3] Gray R.E., Harris G.T. (2019). Renal Cell Carcinoma: Diagnosis and Management. Am. Fam. Physician.

[4] Zerdes I., Tolia M., Tsoukalas N., Mitsis M., Kardamakis D., Pistevou-Gombaki K., Tsekeris P., Kyrgias G. (2019). Systemic therapy of metastatic renal cell carcinoma: Review of the current literature. Urologia.

[5] Singer E.A., Rumble R.B., Van Veldhuizen P.J. (2023). Management of Metastatic Clear Cell Renal Cell Carcinoma: ASCO Guideline Q&A. JCO Oncol. Pract..

[6] Stratigos A.J., Garbe C., Dessinioti C., Lebbe C., van Akkooi A., Bataille V., Bastholt L., Dreno B., Dummer R., Fargnoli M.C. (2023). European consensus-based interdisciplinary guideline for invasive cutaneous squamous cell carcinoma: Part 2. Treatment-Update 2023. Eur. J. Cancer.

[7] National Cancer InstituteSurveillance, Epidemiology, and End Results Program (2023). SEER*Explorer. https://seer.cancer.gov/statistics-network/explorer/.

[8] Schwab M., Hofmann R., Heers H., Hegele A. (2018). mRCC Outcome in the Treatment of Metastatic Renal Cell Carcinoma—A German Single-center Real-world Experience. In Vivo.

[9] Davis I.D., Xie W., Pezaro C., Donskov F., Wells J.C., Agarwal N., Srinivas S., Yuasa T., Beuselinck B., Wood L.A. (2017). Efficacy of Second-line Targeted Therapy for Renal Cell Carcinoma According to Change from Baseline in International Metastatic Renal Cell Carcinoma Database Consortium Prognostic Category. Eur. Urol..

[10] Aldin A., Besiroglu B., Adams A., Monsef I., Piechotta V., Tomlinson E., Hornbach C., Dressen N., Goldkuhle M., Maisch P. (2023). First-line therapy for adults with advanced renal cell carcinoma: A systematic review and network meta-analysis. Cochrane Database Syst. Rev..

[11] Duangthongyou T., Makarasen A., Techasakul S., Chimnoi N., Siripaisarnpipat S. (2011). (-)-crebanine. Acta Crystallogr. Sect. E Struct. Rep. Online.

[12] Tan J., Xiang Y., Xiong Y., Zhang Y., Qiao B., Zhang H. (2023). Crebanine induces ROS-dependent apoptosis in human hepatocellular carcinoma cells via the AKT/FoxO3a signaling pathway. Front. Pharmacol..

[13] Mon M.T., Yodkeeree S., Punfa W., Pompimon W., Limtrakul P. (2018). Alkaloids from Stephania venosa as Chemo-Sensitizers in SKOV3 Ovarian Cancer Cells via Akt/NF-κB Signaling. Chem. Pharm. Bull..

[14] Yodkeeree S., Pompimon W., Limtrakul P. (2014). Crebanine, an aporphine alkaloid, sensitizes TNF-α-induced apoptosis and suppressed invasion of human lung adenocarcinoma cells A549 by blocking NF-κB-regulated gene products. Tumour Biol..

[15] Wongsirisin P., Yodkeeree S., Pompimon W., Limtrakul P. (2012). Induction of G1 Arrest and Apoptosis in Human Cancer Cells by Crebanine, an Alkaloid from *Stephania venosa*. Chem. Pharm. Bull..

[16] Zahari A., Ablat A., Omer N., Nafiah M.A., Sivasothy Y., Mohamad J., Khan M.N., Awang K. (2016). Ultraviolet-visible study on acid-base equilibria of aporphine alkaloids with antiplasmodial and antioxidant activities from *Alseodaphne corneri* and *Dehaasia longipedicellata*. Sci. Rep..

[17] Sun J., Zhan X., Wang W., Yang X., Liu Y., Yang H., Deng J., Yang H. (2024). Natural aporphine alkaloids: A comprehensive review of phytochemistry, pharmacokinetics, anticancer activities, and clinical application. J. Adv. Res..

[18] Krabbe L.M., Margulis V., Lotan Y. (2016). Prognostic Role of Cell Cycle and Proliferative Markers in Clear Cell Renal Cell Carcinoma. Urol. Clin. N. Am..

[19] Wu Y., Terekhanova N.V., Caravan W., Naser Al Deen N., Lal P., Chen S., Mo C.K., Cao S., Li Y., Karpova A. (2023). Epigenetic and transcriptomic characterization reveals progression markers and essential pathways in clear cell renal cell carcinoma. Nat. Commun..

[20] Clark D.J., Dhanasekaran S.M., Petralia F., Pan J., Song X., Hu Y., da Veiga Leprevost F., Reva B., Lih T.M., Chang H.Y. (2019). Integrated Proteogenomic Characterization of Clear Cell Renal Cell Carcinoma. Cell.

[21] Jonasch E., Walker C.L., Rathmell W.K. (2021). Clear cell renal cell carcinoma ontogeny and mechanisms of lethality. Nat. Rev. Nephrol..

[22] Wang J., Su W., Zhang T., Zhang S., Lei H., Ma F., Shi M., Shi W., Xie X., Di C. (2023). Aberrant Cyclin D1 splicing in cancer: From molecular mechanism to therapeutic modulation. Cell Death Dis..

[23] Pistritto G., Trisciuoglio D., Ceci C., Garufi A., D’Orazi G. (2016). Apoptosis as anticancer mechanism: Function and dysfunction of its modulators and targeted therapeutic strategies. Aging.

[24] Carneiro B.A., El-Deiry W.S. (2020). Targeting apoptosis in cancer therapy. Nat. Rev. Clin. Oncol..

[25] Kwon J.W., Oh J.S., Seok S.H., An H.W., Lee Y.J., Lee N.Y., Ha T., Kim H.A., Yoon G.M., Kim S.E. (2023). Combined inhibition of Bcl-2 family members and YAP induces synthetic lethality in metastatic gastric cancer with RASA1 and NF2 deficiency. Mol. Cancer.

[26] Shapiro D.D., Virumbrales-Muñoz M., Beebe D.J., Abel E.J. (2022). Models of Renal Cell Carcinoma Used to Investigate Molecular Mechanisms and Develop New Therapeutics. Front. Oncol..

[27] Lupo B., Trusolino L. (2014). Inhibition of poly(ADP-ribosyl)ation in cancer: Old and new paradigms revisited. Biochim. Biophys. Acta.

[28] Karpova Y., Guo D., Makhov P., Haines A.M., Markov D.A., Kolenko V., Tulin A.V. (2021). Poly(ADP)-Ribosylation Inhibition: A Promising Approach for Clear Cell Renal Cell Carcinoma Therapy. Cancers.

[29] Pletcher J.P., Bhattacharjee S., Doan J.P., Wynn R., Sindhwani P., Nadiminty N., Petros F.G. (2021). The Emerging Role of Poly (ADP-Ribose) Polymerase Inhibitors as Effective Therapeutic Agents in Renal Cell Carcinoma. Front. Oncol..

[30] Elmore S. (2007). Apoptosis: A review of programmed cell death. Toxicol. Pathol..

[31] Slee E.A., Adrain C., Martin S.J. (2001). Executioner caspase-3, -6, and -7 perform distinct, non-redundant roles during the demolition phase of apoptosis. J. Biol. Chem..

[32] Choueiri T.K., Kaelin W.G. (2020). Targeting the HIF2-VEGF axis in renal cell carcinoma. Nat. Med..

[33] Liao C., Hu L., Zhang Q. (2024). Von Hippel-Lindau protein signalling in clear cell renal cell carcinoma. Nat. Rev. Urol..

[34] Milella M., Rutigliano M., Pandolfo S.D., Aveta A., Crocetto F., Ferro M., d’Amati A., Ditonno P., Lucarelli G., Lasorsa F. (2025). The Metabolic Landscape of Cancer Stem Cells: Insights and Implications for Therapy. Cells.

[35] Yoshida Y., Takahashi M., Komine K., Taniguchi S., Yamada H., Sasaki K., Umegaki S., Kawamura Y., Kasahara Y., Ouchi K. (2025). Correlation between Efficacy and Cardiovascular Adverse Events in Patients with Advanced Solid Cancer Who Received VEGF Pathway Inhibitors: Hypertension within the First Eight Weeks Is Associated with Favorable Outcomes of Patients Treated with VEGF Pathway Inhibitors. Intern. Med..

[36] Jin J., Xie Y., Zhang J.-S., Wang J.-Q., Dai S.-J., He W.-f., Li S.-Y., Ashby C.R., Chen Z.-S., He Q. (2023). Sunitinib resistance in renal cell carcinoma: From molecular mechanisms to predictive biomarkers. Drug Resist. Updates.

[37] Powles T., Tomczak P., Park S.H., Venugopal B., Ferguson T., Symeonides S.N., Hajek J., Gurney H., Chang Y.-H., Lee J.L. (2022). Pembrolizumab versus placebo as post-nephrectomy adjuvant therapy for clear cell renal cell carcinoma (KEYNOTE-564): 30-month follow-up analysis of a multicentre, randomised, double-blind, placebo-controlled, phase 3 trial. Lancet Oncol..

[38] Choueiri Toni K., Tomczak P., Park Se H., Venugopal B., Ferguson T., Symeonides Stefan N., Hajek J., Chang Y.-H., Lee J.-L., Sarwar N. (2024). Overall Survival with Adjuvant Pembrolizumab in Renal-Cell Carcinoma. N. Engl. J. Med..

[39] Guo L., An T., Huang Z., Chong T. (2024). A network meta-analysis evaluating the efficacy and safety of adjuvant therapy after nephrectomy in renal cell carcinoma. BMC Urol..

[40] Motzer R., Alekseev B., Rha S.Y., Porta C., Eto M., Powles T., Grünwald V., Hutson T.E., Kopyltsov E., Méndez-Vidal M.J. (2021). Lenvatinib plus Pembrolizumab or Everolimus for Advanced Renal Cell Carcinoma. N. Engl. J. Med..

[41] Motzer R.J., Porta C., Eto M., Powles T., Grünwald V., Hutson T.E., Alekseev B., Rha S.Y., Merchan J., Goh J.C. (2024). Lenvatinib Plus Pembrolizumab Versus Sunitinib in First-Line Treatment of Advanced Renal Cell Carcinoma: Final Prespecified Overall Survival Analysis of CLEAR, a Phase III Study. J. Clin. Oncol..

[42] Motzer R.J., Bex A., Russo P., Tomita Y., Cutuli H.J., Rojas C., Gross-Goupil M., Schinzari G., Melichar B., Barthélémy P. (2024). Adjuvant Nivolumab for Localized Renal Cell Carcinoma at High Risk of Recurrence After Nephrectomy: Part B of the Randomized, Placebo-Controlled, Phase III CheckMate 914 Trial. J. Clin. Oncol..

[43] Garofano G., Saitta C., Musso G., Meagher M.F., Capitanio U., Dabbas M., Birouty N., Karamcheti S., Kim B., Yuen K.L. (2025). Positive Surgical Margins in Clear Cell Renal Cell Carcinoma: Prognostic Impact and Implications for Risk Stratification and Adjuvant Therapy. J. Clin. Med..

[44] Nantapap S., Loetchutinat C., Meepowpan P., Nuntasaen N., Pompimon W. (2010). Antiproliferative Effects of Alkaloids Isolated from the Tuber of Stephania venosa via the Induction of Cell Cycle Arrest in Mammalian Cancer Cell Lines. Am. J. Appl. Sci..

[45] Rose T.L., Kim W.Y. (2024). Renal Cell Carcinoma: A Review. Jama.

[46] Guo Q., Jin Y., Chen X., Ye X., Shen X., Lin M., Zeng C., Zhou T., Zhang J. (2024). NF-κB in biology and targeted therapy: New insights and translational implications. Signal Transduct. Target. Ther..

[47] Greten F.R., Arkan M.C., Bollrath J., Hsu L.C., Goode J., Miething C., Göktuna S.I., Neuenhahn M., Fierer J., Paxian S. (2007). NF-kappaB is a negative regulator of IL-1beta secretion as revealed by genetic and pharmacological inhibition of IKKbeta. Cell.

[48] Wang Y., Lu J., Jiang B., Guo J. (2020). The roles of curcumin in regulating the tumor immunosuppressive microenvironment. Oncol. Lett..

[49] Ebrahimi N., Abdulwahid A.R.R., Mansouri A., Karimi N., Bostani R.J., Beiranvand S., Adelian S., Khorram R., Vafadar R., Hamblin M.R. (2024). Targeting the NF-κB pathway as a potential regulator of immune checkpoints in cancer immunotherapy. Cell Mol. Life Sci..

[50] Xiao Z., Su Z., Han S., Huang J., Lin L., Shuai X. (2020). Dual pH-sensitive nanodrug blocks PD-1 immune checkpoint and uses T cells to deliver NF-κB inhibitor for antitumor immunotherapy. Sci. Adv..

[51] Perez-Ruiz E., Minute L., Otano I., Alvarez M., Ochoa M.C., Belsue V., de Andrea C., Rodriguez-Ruiz M.E., Perez-Gracia J.L., Marquez-Rodas I. (2019). Prophylactic TNF blockade uncouples efficacy and toxicity in dual CTLA-4 and PD-1 immunotherapy. Nature.

[52] Jin X., Ding D., Yan Y., Li H., Wang B., Ma L., Ye Z., Ma T., Wu Q., Rodrigues D.N. (2019). Phosphorylated RB Promotes Cancer Immunity by Inhibiting NF-κB Activation and PD-L1 Expression. Mol. Cell.

[53] Fu M., Hu Y., Lan T., Guan K.L., Luo T., Luo M. (2022). The Hippo signalling pathway and its implications in human health and diseases. Signal Transduct. Target. Ther..

[54] Lv L., Zhou X. (2023). Targeting Hippo signaling in cancer: Novel perspectives and therapeutic potential. MedComm.

[55] Jin X., Zhu L., Xiao S., Cui Z., Tang J., Yu J., Xie M. (2021). MST1 inhibits the progression of breast cancer by regulating the Hippo signaling pathway and may serve as a prognostic biomarker. Mol. Med. Rep..

[56] Yang Y., Hao T., Yao X., Che Y., Liu Y., Fang M., Wang Y., Zhou D., Chai H., Li N. (2023). Crebanine ameliorates ischemia-reperfusion brain damage by inhibiting oxidative stress and neuroinflammation mediated by NADPH oxidase 2 in microglia. Phytomedicine.

[57] Dong S., Ge J., Meng Q., Yuan T., Wang Y., Li Y., Lu Q., Song W., Li Z., Sun S. (2024). Crebanine mitigates glucocorticoid-induced osteonecrosis of the femoral head by restoring bone remodelling homeostasis via attenuating oxidative stress. J. Cell. Mol. Med..

[58] Cui L., Peng C., Li J., Cheng X., Fan X., Li J., Yang Z., Zhao Y., Ma Y. (2022). The anti-inflammatory and analgesic activities of 2Br-Crebanine and Stephanine from Stephania yunnanenses H. S.Lo. Front. Pharmacol..

