# Supplementary material for: Crebanine Induces Cell Death and Alters the Mitotic Process in Renal Cell Carcinoma In Vitro
Source: Int J Mol Sci. 2025 Jul 18;26(14):6896. doi: 10.3390/ijms26146896 (PMC12295564; doi:10.3390/ijms26146896)
Supplement: Supplementary file 1 [file ijms-26-06896-s001.zip › ijms-3726220-supplementary.pdf]

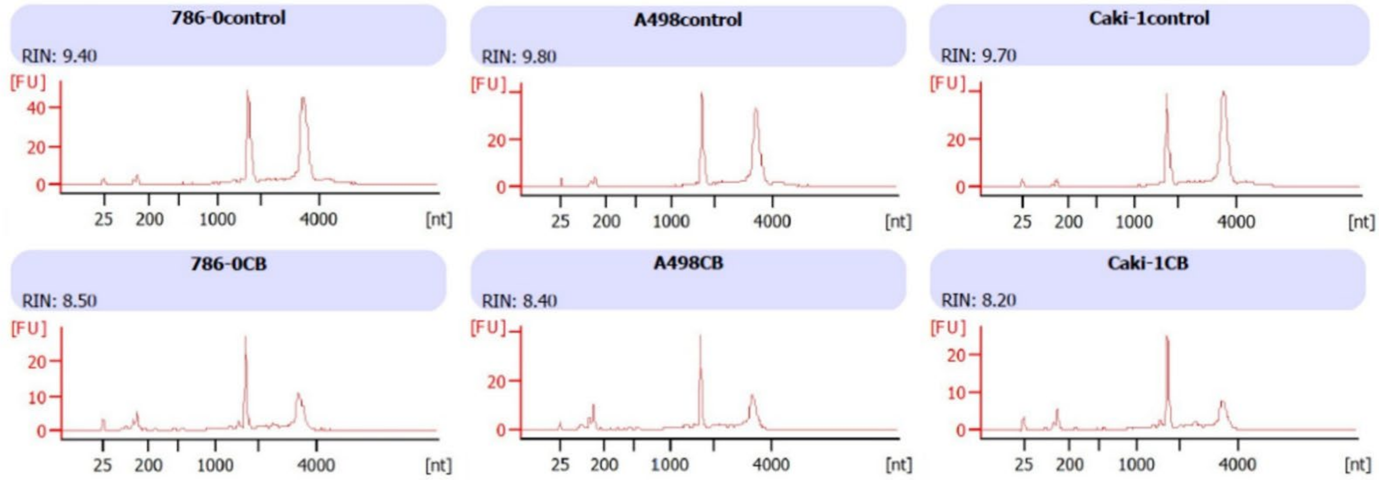

Supplementary Figure S1. The BioAnalyzer2100 electropherograms results of isolated total RNA.

Supplementary Table S1. Sample test results summary table.

Sample name	786-0		A498		Caki-1	
	Control	Crebanine 200 μM	Control	Crebanine 200 μM	Control	Crebanine 200 μM
Quantitative analysis						
Concentration (ng/μl)	17572	308	3276	448	14739	250
Volume (μl)	25	25	25	25	25	25
Total amount (μg)	172.44	5.17	48.53	6.62	190.06	5.07
OD260/OD280	2.08	2.05	2.03	2.03	2.07	2.05
OD260 /OD230	2.18	0.69	2.14	1.33	2.18	1.38
Qualitative analysis						
18S/28S (16S/23S)	1.8	1	1.8	1.1	1.9	0.8
RNA Integrity Number	9.4	8.5	9.8	8.4	9.7	8.2

Supplementary Table S2. Raw RNA-seq data of all differentially expressed genes.

Geneid	Symbol	log_2FC	*p* value	*adjusted p-value*
ENSG00000130402	ACTN4	-2.22	1.1E-20	2.18039E-16
ENSG00000099204	ABLIM1	-2.22	2.4E-17	2.31921E-13
ENSG00000175197	DDIT3	5.26	5.8E-17	3.70605E-13
ENSG00000065534	MYLK	-3.19	9.7E-17	4.64702E-13
ENSG00000068654	POLR1A	-2.64	3.7E-16	1.42599E-12
ENSG00000152463	OLAH	6.27	2.2E-15	7.11467E-12
ENSG00000163485	ADORA1	-4.50	5.1E-15	1.39198E-11
ENSG00000133639	BTG1	2.71	7.5E-15	1.75381E-11
ENSG00000151743	AMN1	3.06	8.2E-15	1.75381E-11
ENSG00000240184	PCDHGC3	-3.74	1.2142E-14	2.13889E-11
ENSG00000095319	NUP188	-2.1068281	1.22229E-14	2.13889E-11
ENSG00000136731	UGGT1	-2.782108208	4.1552E-14	6.66529E-11
ENSG00000127616	SMARCA4	-2.58145037	4.7982E-14	7.10466E-11
ENSG00000067798	NAV3	-3.772685271	8.03835E-14	1.03153E-10
ENSG00000172748	ZNF596	3.465629717	9.2565E-14	1.11362E-10
ENSG00000211459	RNR1	4.044842324	4.64692E-13	5.26168E-10
ENSG00000210112	TRNM	5.238424093	5.45022E-13	5.8284E-10
ENSG00000013441	CLK1	3.065357811	8.27478E-13	8.38322E-10
ENSG00000108312	UBTF	-2.186984697	1.4447E-12	1.39045E-09
ENSG00000174332	GLIS1	-3.519642736	3.68172E-12	3.37473E-09
ENSG00000210107	TRNQ	-3.938639265	4.56205E-12	3.84507E-09
ENSG00000114784	EIF1B	2.643297608	4.59435E-12	3.84507E-09
ENSG00000018408	WWTR1	-2.234820747	5.27985E-12	4.23466E-09
ENSG00000187498	COL4A1	-3.424943384	5.58419E-12	4.2996E-09

ENSG00000185250	PPIL6	3.006031311	7.19421E-12	5.3262E-09
ENSG00000173812	EIF1	2.067178009	1.39396E-11	9.93792E-09
ENSG00000286546	LOC613266	-6.298395989	1.55257E-11	1.06734E-08
ENSG00000074590	NUAK1	-2.231581745	2.03971E-11	1.35387E-08
ENSG00000188243	COMMD6	2.444304084	2.18802E-11	1.40391E-08
ENSG00000111897	SERINC1	2.046490195	2.47537E-11	1.53704E-08
ENSG00000275410	HNF1B	-3.240679645	4.97343E-11	2.99168E-08
ENSG00000144642	RBMS3	-2.077587761	5.46774E-11	3.18935E-08
ENSG00000163393	SLC22A15	2.370310948	6.83524E-11	3.86975E-08
ENSG00000228544	CCDC183-AS1	-4.438999617	1.16185E-10	6.21234E-08
ENSG00000091106	NLRC4	10.10208835	1.26036E-10	6.55691E-08
ENSG00000090530	P3H2	-2.99603439	1.34181E-10	6.79699E-08
ENSG00000121749	TBC1D15	2.847376501	1.55675E-10	7.62627E-08
ENSG00000105997	HOXA3	-4.03988379	1.58476E-10	7.62627E-08
ENSG00000261115	TMEM178B	-3.955329117	1.90054E-10	8.92282E-08
ENSG00000186575	NF2	-2.287498495	2.36341E-10	1.08317E-07
ENSG00000186666	BCDIN3D	2.464647334	2.47202E-10	1.1066E-07
ENSG00000179044	EXOC3L1	5.031026773	3.54804E-10	1.51769E-07
ENSG00000133056	PIK3C2B	-3.037052085	5.03763E-10	2.10534E-07
ENSG00000115526	CHST10	-2.231581952	5.14059E-10	2.10534E-07
ENSG00000178202	POGLUT3	-3.119751542	5.41968E-10	2.1734E-07
ENSG00000072310	SREBF1	-2.287526812	5.60765E-10	2.20289E-07
ENSG00000104356	POP1	-2.729881689	6.05727E-10	2.33193E-07
ENSG00000203668	CHML	-2.370508969	6.4051E-10	2.41749E-07
ENSG00000167889	MGAT5B	-4.315665186	7.21902E-10	2.67229E-07
ENSG00000258102	MAP1LC3B2	3.223483851	7.89657E-10	2.86794E-07
ENSG00000170634	ACYP2	2.716840231	1.12695E-09	4.01717E-07
ENSG00000165572	KBTBD6	-2.34175427	1.21405E-09	4.24897E-07
ENSG00000002746	HECW1	-2.612031633	1.35907E-09	4.59136E-07
ENSG00000107331	ABCA2	-2.793043257	1.35959E-09	4.59136E-07
ENSG00000235385	LINC02154	5.493810665	1.40593E-09	4.66598E-07
ENSG00000210100	TRNI	3.986877277	1.43767E-09	4.69046E-07
ENSG00000163536	SERPINI1	4.224111335	1.62111E-09	5.11553E-07
ENSG00000140941	MAP1LC3B	3.179206312	1.96969E-09	6.01818E-07
ENSG00000157657	ZNF618	-3.743193246	2.08124E-09	6.25965E-07
ENSG00000204362	LINC02783	-4.086013455	2.18464E-09	6.46957E-07
ENSG00000176473	WDR25	2.04156287	2.22046E-09	6.476E-07
ENSG00000163362	INAVA	-3.490819894	2.53951E-09	7.18868E-07
ENSG00000166897	ELFN2	-3.883749078	2.8248E-09	7.88036E-07
ENSG00000058056	USP13	-3.862946948	3.10463E-09	8.41705E-07
ENSG00000183048	SLC25A10	-3.994780029	3.2832E-09	8.77754E-07
ENSG00000087074	PPP1R15A	2.973974294	4.2929E-09	1.13197E-06
ENSG00000179841	AKAP5	-3.695062089	5.45804E-09	1.41744E-06
ENSG00000254726	MEX3A	-3.343860032	5.5228E-09	1.41744E-06

ENSG00000234741	GAS5	3.122142971	5.99799E-09	1.48477E-06
ENSG00000111602	TIMELESS	-2.409179372	6.86743E-09	1.65239E-06
ENSG00000127603	MACF1	-2.327985054	7.05598E-09	1.6768E-06
ENSG00000232499	LOC100421402	-2.411894777	7.61938E-09	1.77493E-06
ENSG00000139973	SYT16	-4.033715366	7.65333E-09	1.77493E-06
ENSG00000106785	TRIM14	-4.156188695	7.91471E-09	1.81369E-06
ENSG00000186469	GNG2	-3.346472298	1.0054E-08	2.19955E-06
ENSG00000111077	TNS2	-2.788356973	1.01285E-08	2.19955E-06
ENSG00000125966	MMP24	-4.745577454	1.01699E-08	2.19955E-06
ENSG00000210194	TRNE	-3.459524333	1.04692E-08	2.23913E-06
ENSG00000173110	HSPA6	6.190483401	1.07588E-08	2.26588E-06
ENSG00000276043	UHRF1	-5.052200424	1.08297E-08	2.26588E-06
ENSG00000111644	ACRBP	5.923466254	1.19758E-08	2.47873E-06
ENSG00000136205	TNS3	-3.057766282	1.56174E-08	3.09986E-06
ENSG00000154127	UBASH3B	-3.004291675	1.80248E-08	3.54041E-06
ENSG00000070669	ASNS	3.453464272	1.85372E-08	3.58425E-06
ENSG00000221890	NPTXR	-3.20040767	1.86553E-08	3.58425E-06
ENSG00000259674	RPL7AP75	-5.709728817	1.88066E-08	3.58425E-06
ENSG00000185379	RAD51D	-2.51691133	1.95952E-08	3.69792E-06
ENSG00000104835	SARS2	-3.159975553	2.4519E-08	4.53813E-06
ENSG00000171793	CTPS1	-2.735853999	2.64002E-08	4.79413E-06
ENSG00000115221	ITGB6	-5.440527497	2.72276E-08	4.88083E-06
ENSG00000285972	CERNA2	-3.720886209	2.73848E-08	4.88083E-06
ENSG00000117614	SYF2	2.252322465	2.957E-08	5.22195E-06
ENSG00000172009	THOP1	-2.53267887	3.32349E-08	5.77582E-06
ENSG00000167972	ABCA3	2.703091929	3.33064E-08	5.77582E-06
ENSG00000197385	ZNF860	-3.240674377	3.74994E-08	6.34789E-06
ENSG00000026036	RTEL1-TNFRSF6B	-4.878534181	3.75946E-08	6.34789E-06
ENSG00000075624	ACTB	-2.314885741	3.93292E-08	6.52628E-06
ENSG00000183696	UPP1	3.298881649	4.60497E-08	7.44884E-06
ENSG00000198796	ALPK2	-3.097697677	4.73495E-08	7.59526E-06
ENSG00000128731	HERC2	-2.151177609	6.44679E-08	1.0089E-05
ENSG00000260454	LINC02957	6.534694724	6.50757E-08	1.01019E-05
ENSG00000113273	ARSB	-2.632460856	6.64794E-08	1.02373E-05
ENSG00000145777	TSLP	5.445990859	6.86583E-08	1.04067E-05
ENSG00000143799	PARP1	-2.023598869	6.86609E-08	1.04067E-05
ENSG00000143321	HDGF	-2.081554138	6.98682E-08	1.0507E-05
ENSG00000143341	HMCN1	-2.399497522	7.27649E-08	1.08578E-05
ENSG00000198556	ZNF789	-2.380402487	7.78438E-08	1.15263E-05
ENSG00000095209	TMEM38B	2.110255627	8.23429E-08	1.20994E-05
ENSG00000232671	ZNF687-AS1	2.848536604	8.34009E-08	1.2162E-05
ENSG00000172264	MACROD2	-3.604565612	9.27775E-08	1.34276E-05
ENSG00000121236	TRIM6	-2.931156578	1.05024E-07	1.50867E-05

ENSG00000177954	RPS27	2.135538067	1.12346E-07	1.60188E-05
ENSG00000186132	C2orf76	2.965993663	1.13814E-07	1.61089E-05
ENSG00000115419	GLS	-2.328713979	1.18513E-07	1.64528E-05
ENSG00000174130	TLR6	-2.459620848	1.21272E-07	1.6564E-05
ENSG00000234685	NUS1P2	-3.578450374	1.21332E-07	1.6564E-05
ENSG00000138495	COX17	2.662696453	1.27498E-07	1.72831E-05
ENSG00000169851	PCDH7	-3.140626409	1.35558E-07	1.82472E-05
ENSG00000119048	UBE2B	2.222102723	1.37151E-07	1.83335E-05
ENSG00000189223	PAX8-AS1	5.01770308	1.4273E-07	1.88179E-05
ENSG00000137502	RAB30	-2.226201897	1.44175E-07	1.88791E-05
ENSG00000196155	PLEKHG4	-3.061194563	1.46684E-07	1.90779E-05
ENSG00000273899	NOL12	-3.238383039	1.49272E-07	1.92841E-05
ENSG00000120800	UTP20	-2.090465653	1.65357E-07	2.12197E-05
ENSG00000176619	LMNB2	-2.972307083	1.82997E-07	2.31744E-05
ENSG00000140598	EFL1	2.427304317	1.87323E-07	2.35672E-05
ENSG00000083444	PLOD1	-2.006529731	1.92432E-07	2.40527E-05
ENSG00000155666	KDM8	-3.06514953	1.95668E-07	2.42994E-05
ENSG00000117009	KMO	-5.422531358	2.08564E-07	2.5735E-05
ENSG00000165376	CLDN2	-5.21220834	2.13523E-07	2.6179E-05
ENSG00000213937	CLDN9	5.068911704	2.16475E-07	2.6373E-05
ENSG00000234616	JRK	-2.607238413	2.22005E-07	2.68766E-05
ENSG00000157796	WDR19	2.245801033	2.26221E-07	2.72158E-05
ENSG00000260804	LINC01963	-3.166162832	2.29038E-07	2.73836E-05
ENSG00000113249	HAVCR1	-3.725378157	2.36686E-07	2.81233E-05
ENSG00000276600	RAB7B	-6.654308973	2.48508E-07	2.93468E-05
ENSG00000187808	SOWAHD	-5.754864231	2.67224E-07	3.13646E-05
ENSG00000024862	CCDC28A	2.190870908	2.73756E-07	3.19366E-05
ENSG00000006652	IFRD1	2.18652855	2.76943E-07	3.21137E-05
ENSG00000073711	PPP2R3A	-2.142040144	2.93369E-07	3.36135E-05
ENSG00000213300	HNRNPA3P6	-2.339479133	3.51493E-07	3.97993E-05
ENSG00000155254	MARVELD1	-2.593200977	3.69636E-07	4.16089E-05
ENSG00000220804	LINC01881	2.349952814	3.72351E-07	4.16708E-05
ENSG00000138642	HERC6	-2.91670704	3.85954E-07	4.29435E-05
ENSG00000182179	UBA7	-2.694960531	3.94181E-07	4.35633E-05
ENSG00000137393	RNF144B	-2.970835015	3.9605E-07	4.35633E-05
ENSG00000259583	ALDH1A3-AS1	-3.754963415	4.12389E-07	4.51028E-05
ENSG00000165312	OTUD1	2.312120763	4.27813E-07	4.65253E-05
ENSG00000273061	CDC37L1-DT	3.614863145	4.4245E-07	4.75794E-05
ENSG00000172901	LVRN	4.554536056	4.57154E-07	4.88875E-05
ENSG00000108509	CAMTA2	2.102154109	4.96571E-07	5.19484E-05
ENSG00000116668	SWT1	2.113525819	5.14953E-07	5.30071E-05
ENSG00000228393	LINC01004	2.676270358	5.24875E-07	5.3741E-05
ENSG00000223891	OSER1-DT	2.941683314	5.31426E-07	5.41239E-05
ENSG00000122870	BICC1	-2.683465639	5.52828E-07	5.57141E-05

ENSG00000167964	RAB26	-3.379462073	5.6186E-07	5.6303E-05
ENSG00000272325	NUDT3	-2.37283797	5.64521E-07	5.6303E-05
ENSG00000127129	EDN2	-6.890374441	5.68508E-07	5.64083E-05
ENSG00000154237	LRRK1	-2.746876848	6.0369E-07	5.92879E-05
ENSG00000166670	MMP10	7.977180558	6.3927E-07	6.24635E-05
ENSG00000128602	SMO	-3.397792507	6.51496E-07	6.33366E-05
ENSG00000137434	C6orf52	4.192044897	7.0123E-07	6.74898E-05
ENSG00000162772	ATF3	4.61465633	7.26514E-07	6.9231E-05
ENSG00000120075	HOXB5	-3.011754529	7.44941E-07	7.0291E-05
ENSG00000253773	CFAP418-AS1	7.764148672	7.74326E-07	7.27073E-05
ENSG00000204371	EHMT2	-2.113180209	8.28593E-07	7.70511E-05
ENSG00000101049	SGK2	-3.326331501	9.34381E-07	8.6057E-05
ENSG00000135697	BCO1	-5.585550202	9.53597E-07	8.74085E-05
ENSG00000266074	BAHCC1	-2.791544257	1.1564E-06	0.000102108
ENSG00000260708	TBC1D22A-DT	2.419132653	1.16807E-06	0.000102667
ENSG00000146469	VIP	6.255813267	1.23262E-06	0.000107361
ENSG00000166173	LARP6	2.201336484	1.36854E-06	0.000117309
ENSG00000171631	P2RY6	-3.99398963	1.39398E-06	0.000118729
ENSG00000196123	MATCAP1	-2.20634794	1.43217E-06	0.000121157
ENSG00000132688	NES	-3.703710948	1.43508E-06	0.000121157
ENSG00000136295	TTYH3	-2.55903051	1.44476E-06	0.000121442
ENSG00000157600	TMEM164	-2.03598947	1.51428E-06	0.000126183
ENSG00000175311	ANKS4B	5.075569847	1.53011E-06	0.000126953
ENSG00000188549	CCDC9B	-3.044947388	1.58008E-06	0.000129128
ENSG00000171791	BCL2	-2.373196376	1.58315E-06	0.000129128
ENSG00000166938	DIS3L	-2.101050046	1.67679E-06	0.000136188
ENSG00000148835	TAF5	2.070367729	1.7243E-06	0.000138851
ENSG00000139318	DUSP6	2.986653355	1.73122E-06	0.000138851
ENSG00000113389	NPR3	4.602957406	1.85193E-06	0.000146798
ENSG00000003096	KLHL13	-2.192404128	2.0755E-06	0.000163235
ENSG00000076248	UNG	-2.20681433	2.07794E-06	0.000163235
ENSG00000168916	ZNF608	-2.476300636	2.08613E-06	0.000163235
ENSG00000198695	ND6	-2.632277202	2.13984E-06	0.000166159
ENSG00000149177	PTPRJ	-2.028297386	2.26884E-06	0.000173995
ENSG00000188827	SLX4	-2.234955029	2.35858E-06	0.000179775
ENSG00000125618	PAX8	-3.030216995	2.37222E-06	0.000179775
ENSG00000261087	ZNNT1	3.046249576	2.51819E-06	0.000190089
ENSG00000176046	NUPR1	2.554287568	2.55895E-06	0.000192411
ENSG00000046889	PREX2	-2.684002771	2.81177E-06	0.000208169
ENSG00000258183	LINC02392	6.24099046	2.845E-06	0.000209021
ENSG00000257851	HNRNPA3P10	-2.471123841	3.0344E-06	0.000220412
ENSG00000229474	PATL2	3.462573408	3.21237E-06	0.000229018
ENSG00000120318	ARAP3	-2.214313997	3.27546E-06	0.000231799
ENSG00000225345	SNX18P3	-2.660298639	3.32597E-06	0.000233655

ENSG00000100065	CARD10	-2.886482351	3.34079E-06	0.000233843
ENSG00000143850	PLEKHA6	-2.272309842	3.37522E-06	0.000235397
ENSG00000115107	STEAP3	-2.843521736	3.54227E-06	0.000245212
ENSG00000147894	C9orf72	2.55756933	3.55416E-06	0.000245212
ENSG00000176273	SLC35G1	-2.835675265	3.57859E-06	0.000246015
ENSG00000164266	SPINK1	4.714066719	3.69174E-06	0.000252891
ENSG00000234707	SEC61G-DT	6.238404413	3.70565E-06	0.000252943
ENSG00000262001	DLGAP1-AS2	3.746886538	3.87307E-06	0.000260674
ENSG00000273079	GRIN2B	-4.479378244	4.28171E-06	0.00028523
ENSG00000185215	TNFAIP2	-2.948958039	4.28238E-06	0.00028523
ENSG00000140263	SORD	-2.299554997	4.3163E-06	0.000286498
ENSG00000169213	RAB3B	-2.61442086	4.48586E-06	0.000294704
ENSG00000138496	PARP9	-2.099127123	4.52804E-06	0.000296145
ENSG00000231574	LINC02015	-4.090909176	4.67465E-06	0.000303994
ENSG00000139531	SUOX	-2.187236099	4.72435E-06	0.000306192
ENSG00000162144	CYB561A3	-2.046882314	4.84774E-06	0.000313135
ENSG00000166278	C2	3.332989084	5.29695E-06	0.000338741
ENSG00000188060	RAB42	-2.576471684	5.43482E-06	0.000345264
ENSG00000157343	ARMC12	3.574964036	5.45677E-06	0.000345518
ENSG00000111331	OAS3	-3.429625571	5.50706E-06	0.000347559
ENSG00000246334	PRR7-AS1	-3.641708348	5.55823E-06	0.000349642
ENSG00000214353	VAC14-AS1	-6.259295019	5.68384E-06	0.000356378
ENSG00000100490	CDKL1	-2.005360984	5.89166E-06	0.000367018
ENSG00000241635	UGT1A1	-4.358039752	5.95117E-06	0.000368781
ENSG00000110944	IL23A	3.740563656	5.95828E-06	0.000368781
ENSG00000006025	OSBPL7	-2.869036176	6.09972E-06	0.000376326
ENSG00000197889	MEIG1	3.473606546	6.19973E-06	0.000380284
ENSG00000100557	CCDC198	-4.280853163	6.24291E-06	0.000380284
ENSG00000064393	HIPK2	-2.500843984	6.31548E-06	0.000383491
ENSG00000257122	RRN3P3	-2.701748691	6.37688E-06	0.000386002
ENSG00000184384	MAML2	-2.328966618	6.43877E-06	0.000388122
ENSG00000162032	SPSB3	2.191768182	6.52236E-06	0.000389904
ENSG00000118257	NRP2	-2.213864042	6.69327E-06	0.000396006
ENSG00000151892	GFRA1	-3.697368109	6.73187E-06	0.000396006
ENSG00000188672	RHCE	4.651265316	6.85345E-06	0.000400979
ENSG00000251602	MTA1-DT	-3.57039405	6.94644E-06	0.000403742
ENSG00000168936	TMEM129	-2.377239331	7.07482E-06	0.000407734
ENSG00000185432	METTL7A	-6.467146613	7.10414E-06	0.000408202
ENSG00000038427	VCAN	-3.563905522	7.15763E-06	0.000410051
ENSG00000260083	MIR762HG	2.194846332	7.34012E-06	0.000418017
ENSG00000165804	ZNF219	-2.058581405	7.5848E-06	0.000425503
ENSG00000113924	HGD	-3.1440412	7.59667E-06	0.000425503
ENSG00000039139	DNAH5	-2.362224264	7.94147E-06	0.000441807
ENSG00000108797	CNTNAP1	-2.110604746	8.00843E-06	0.000444249

ENSG00000164746	C7orf57	2.620320329	8.13386E-06	0.000449037
ENSG00000173212	MAB21L3	-3.361149655	8.24799E-06	0.000453616
ENSG00000248489	LOC100289230	3.318321273	8.47173E-06	0.000464594
ENSG00000214717	ZBED1	-2.0183397	8.55666E-06	0.000464875
ENSG00000182600	SNORC	2.335167835	9.42909E-06	0.000506985
ENSG00000210082	RNR2	2.275956959	9.54799E-06	0.000511948
ENSG00000161048	NAPEPLD	-3.703282423	9.7521E-06	0.000518086
ENSG00000166340	TPP1	2.099427665	1.02255E-05	0.000534865
ENSG00000204334	ERICH2	3.201818019	1.02639E-05	0.000535419
ENSG00000205978	NYNRIN	-3.44927311	1.03264E-05	0.000535861
ENSG00000231770	TMEM44-AS1	2.29330107	1.03544E-05	0.000535861
ENSG00000204531	POU5F1	-2.527864821	1.03855E-05	0.000535861
ENSG00000174827	PDZK1	-5.086908126	1.04116E-05	0.000535861
ENSG00000180089	TMEM86B	2.511503923	1.04762E-05	0.000536478
ENSG00000196482	ESRRG	-3.607882068	1.07079E-05	0.000545018
ENSG00000125872	LRRN4	-5.714928392	1.07582E-05	0.000545018
ENSG00000062282	DGAT2	-2.7175197	1.07594E-05	0.000545018
ENSG00000108439	PNPO	-2.157312687	1.17514E-05	0.000587538
ENSG00000079482	OPHN1	-2.164808653	1.191E-05	0.000593926
ENSG00000010704	HFE	-2.777525255	1.2361E-05	0.000613238
ENSG00000182054	IDH2	-2.825600824	1.24038E-05	0.000613746
ENSG00000134247	PTGFRN	-2.913495503	1.2435E-05	0.000613746
ENSG00000177409	SAMD9L	-3.137710572	1.33206E-05	0.000650783
ENSG00000162390	ACOT11	-5.127374395	1.3482E-05	0.000657001
ENSG00000185022	MAFF	2.564646385	1.3917E-05	0.00067478
ENSG00000134215	VAV3	-3.170421665	1.40207E-05	0.000676402
ENSG00000245748	LOC100129931	-6.378317469	1.44461E-05	0.000695184
ENSG00000172361	CFAP53	2.556674178	1.48847E-05	0.000710957
ENSG00000177548	RABEP2	-2.436341505	1.54087E-05	0.000730549
ENSG00000259291	ZNF710-AS1	-3.888975027	1.55645E-05	0.000736122
ENSG00000159588	CCDC17	3.962768979	1.63419E-05	0.000767231
ENSG00000100344	PNPLA3	-2.308464932	1.65857E-05	0.000776784
ENSG00000151474	FRMD4A	-2.85913906	1.66822E-05	0.000779407
ENSG00000135124	P2RX4	2.009820353	1.74468E-05	0.000813155
ENSG00000253293	HOXA10	-2.360533011	1.77585E-05	0.000825686
ENSG00000181016	LSMEM1	3.46342282	1.78966E-05	0.000830099
ENSG00000114805	PLCH1	-3.385800705	1.83202E-05	0.000845674
ENSG00000115602	IL1RL1	6.955369047	1.87087E-05	0.000857478
ENSG00000260317	LINC02986	3.474265346	1.91654E-05	0.000874205
ENSG00000162407	PLPP3	-2.056613213	1.92285E-05	0.000875011
ENSG00000196196	HRCT1	-4.484663388	1.93683E-05	0.000879292
ENSG00000188582	PAQR9	-4.239346983	1.94987E-05	0.000881441
ENSG00000204923	FBXO48	2.176367329	1.95072E-05	0.000881441
ENSG00000151150	ANK3	-4.476607018	1.96311E-05	0.000884964

ENSG00000179454	KLHL28	2.236171861	1.97801E-05	0.000889597
ENSG00000224032	EPB41L4A-AS1	2.238116025	1.98518E-05	0.000890738
ENSG00000113645	WWC1	-2.025973515	2.08938E-05	0.00092968
ENSG00000131389	SLC6A6	-2.142544975	2.12238E-05	0.000937045
ENSG00000007968	E2F2	-4.328286405	2.12871E-05	0.000937045
ENSG00000271869	DCTN6-DT	2.652623574	2.13219E-05	0.000937045
ENSG00000087077	TRIP6	-2.064521649	2.1824E-05	0.000948284
ENSG00000108474	PIGL	2.090003846	2.24427E-05	0.000970787
ENSG00000106006	HOXA6	-2.302526521	2.26241E-05	0.000974253
ENSG00000103044	HAS3	-3.043141242	2.32981E-05	0.000994378
ENSG00000164463	CREBRF	2.841130258	2.34296E-05	0.000996148
ENSG00000165521	EML5	3.30105678	2.45794E-05	0.001035293
ENSG00000070614	NDST1	-2.723201361	2.47773E-05	0.001037851
ENSG00000143195	ILDR2	-5.782853596	2.48019E-05	0.001037851
ENSG00000213888	LINC01521	-3.507888432	2.51287E-05	0.001044713
ENSG00000177410	ZFAS1	2.347871771	2.84615E-05	0.001158257
ENSG00000158089	GALNT14	-2.321486236	2.89574E-05	0.001171012
ENSG00000197410	DCHS2	-3.454164231	2.95191E-05	0.001186248
ENSG00000143409	MINDY1	-2.275570067	2.98316E-05	0.001193822
ENSG00000205853	RFPL3S	3.221126466	2.99305E-05	0.001195295
ENSG00000258366	RTEL1	-3.907613068	3.03068E-05	0.001206831
ENSG00000210117	TRNW	2.981583069	3.03448E-05	0.001206831
ENSG00000214381	LINC00488	-5.661289026	3.05507E-05	0.001212517
ENSG00000172183	ISG20	2.489174143	3.08574E-05	0.001217162
ENSG00000253729	PRKDC	-2.223622946	3.09439E-05	0.001218074
ENSG00000158270	COLEC12	4.373011472	3.10741E-05	0.001220706
ENSG00000182263	FIGN	-2.162255373	3.13604E-05	0.001227549
ENSG00000138061	CYP1B1	-3.188640374	3.14525E-05	0.00122805
ENSG00000255346	NOX5	-3.989086435	3.17895E-05	0.001238698
ENSG00000256128	LINC00944	2.35194283	3.25391E-05	0.001262792
ENSG00000116761	CTH	2.410587758	3.45562E-05	0.001325045
ENSG00000157193	LRP8	-2.581147795	3.6644E-05	0.001391242
ENSG00000166341	DCHS1	-2.914825602	3.67667E-05	0.001393156
ENSG00000213057	C1orf220	-4.212992635	3.8329E-05	0.001438197
ENSG00000117228	GBP1	-3.174295454	3.85995E-05	0.00144259
ENSG00000163701	IL17RE	3.248065394	3.86709E-05	0.00144259
ENSG00000230453	ANKRD18B	-2.184161865	3.89832E-05	0.001448624
ENSG00000244509	APOBEC3C	-2.205932336	3.9812E-05	0.001476574
ENSG00000149596	JPH2	-2.685884164	3.98909E-05	0.001476655
ENSG00000126351	THRA	-2.352711263	4.0295E-05	0.001485899
ENSG00000062524	LTK	5.661239954	4.04341E-05	0.001486646
ENSG00000126368	NR1D1	2.67608016	4.0658E-05	0.001490717
ENSG00000198053	SIRPA	-3.0106601	4.08609E-05	0.001495308
ENSG00000167900	TK1	-2.264016375	4.10299E-05	0.001495805

ENSG00000258791	LINC00520	6.377606236	4.1439E-05	0.001505017
ENSG00000164125	GASK1B	-3.8530397	4.18217E-05	0.001513208
ENSG00000270959	LPP-AS2	-3.177128805	4.1949E-05	0.001514963
ENSG00000281398	SNHG4	-2.126253423	4.21598E-05	0.001518767
ENSG00000161558	TMEM143	-2.024884079	4.22567E-05	0.001518767
ENSG00000179148	ALOXE3	2.674826243	4.23233E-05	0.001518767
ENSG00000151388	ADAMTS12	-3.705530208	4.27074E-05	0.001528021
ENSG00000100105	PATZ1	-2.119928966	4.30776E-05	0.001538407
ENSG00000224165	DNAJC27-AS1	3.406986282	4.33223E-05	0.001540884
ENSG00000123080	CDKN2C	-2.427642555	4.33871E-05	0.001540884
ENSG00000272269	NUP153-AS1	-2.280548991	4.405E-05	0.001555813
ENSG00000153086	ACMSD	-4.531726192	4.4945E-05	0.001584517
ENSG00000169683	LRRC45	-2.241850115	4.50732E-05	0.001586132
ENSG00000123570	RAB9B	-2.002135741	4.5216E-05	0.001588255
ENSG00000113525	IL5	4.626729295	4.63096E-05	0.001623703
ENSG00000226562	CYP4F26P	-2.874767784	4.75867E-05	0.001647477
ENSG00000176974	SHMT1	-2.40097284	4.98077E-05	0.001712051
ENSG00000162337	LRP5	-2.396195562	5.02013E-05	0.001722504
ENSG00000230424	EMC1-AS1	3.10671606	5.09528E-05	0.00174518
ENSG00000101265	RASSF2	-3.075001428	5.26604E-05	0.00179092
ENSG00000142798	HSPG2	-3.284939574	5.29062E-05	0.001794639
ENSG00000127325	BEST3	-4.337847093	5.29563E-05	0.001794639
ENSG00000225329	LHFPL3-AS2	4.903118062	5.34973E-05	0.001806613
ENSG00000142619	PADI3	-4.54388938	5.56163E-05	0.00186834
ENSG00000257433	RPAP3-DT	2.371060001	5.62859E-05	0.001887538
ENSG00000137404	NRM	-2.24547756	5.65527E-05	0.001893188
ENSG00000162745	OLFML2B	-3.689575244	5.68817E-05	0.001900894
ENSG00000120656	TAF12	2.120843624	5.71123E-05	0.001905294
ENSG00000213901	SLC23A3	2.49273922	5.91672E-05	0.001963637
ENSG00000168874	ATOH8	-4.125431034	5.98427E-05	0.001982637
ENSG00000228606	DCAF8-DT	3.190966806	6.31165E-05	0.002066205
ENSG00000146278	PNRC1	2.52405956	6.35216E-05	0.002075819
ENSG00000168077	SCARA3	-3.028218109	6.36258E-05	0.002075819
ENSG00000163762	TM4SF18	-4.44056754	6.48838E-05	0.00210971
ENSG00000075275	CELSR1	-2.134200872	6.57582E-05	0.002130943
ENSG00000139211	AMIGO2	-2.369230856	6.72693E-05	0.002168957
ENSG00000109063	MYH3	2.979140944	6.85361E-05	0.002198754
ENSG00000109083	IFT20	2.016667624	6.86792E-05	0.002199676
ENSG00000169826	CSGALNACT2	2.284519206	6.9745E-05	0.002224025
ENSG00000197748	CFAP43	2.133503577	7.00107E-05	0.002224025
ENSG00000184227	ACOT1	-2.294899578	7.02482E-05	0.002224025
ENSG00000171617	ENC1	-2.345793854	7.10156E-05	0.002240951
ENSG00000205436	EXOC3L4	-4.956443187	7.20261E-05	0.002269116
ENSG00000286369	LOC105369363	4.513280729	7.34319E-05	0.002302102

ENSG00000099250	NRP1	-2.20696487	7.3798E-05	0.002309817
ENSG00000160867	FGFR4	-2.00985541	7.52651E-05	0.002344301
ENSG00000099338	CATSPERG	3.634433288	7.98906E-05	0.002446768
ENSG00000105855	ITGB8	-3.159743832	8.16306E-05	0.002479708
ENSG00000151612	ZNF827	-2.279903793	8.33377E-05	0.002522276
ENSG00000113492	AGXT2	-4.423192996	8.39209E-05	0.002533268
ENSG00000065057	NTHL1	-2.13278813	8.76358E-05	0.002611302
ENSG00000287382	LOC105377663	2.876475114	8.84885E-05	0.002624249
ENSG00000188015	S100A3	-2.550152139	8.96478E-05	0.002646674
ENSG00000184985	SORCS2	-2.763315009	9.33728E-05	0.00273151
ENSG00000230797	YY2	-2.181390678	9.53988E-05	0.002782321
ENSG00000155749	FLACC1	3.256594323	9.60372E-05	0.002788265
ENSG00000115318	LOXL3	-2.973713753	9.71951E-05	0.002802816
ENSG00000197520	FAM177B	5.03298715	9.80163E-05	0.002820204
ENSG00000073111	MCM2	-2.029829936	9.85881E-05	0.002832341
ENSG00000165029	ABCA1	-2.058230224	0.000100011	0.002860505
ENSG00000253187	HOXA10-AS	-2.472016421	0.00010054	0.002871361
ENSG00000113811	SELENOK	2.047150246	0.000100865	0.002872123
ENSG00000188707	ZBED10P	-3.886413226	0.000103897	0.002936726
ENSG00000178921	PFAS	-2.324334214	0.00010518	0.00295564
ENSG00000223703	IGSF3P2	-6.486494554	0.000106725	0.002981629
ENSG00000257732	LOC124903002	2.786031768	0.000107826	0.002996221
ENSG00000257642	C12orf75-AS1	-2.593450163	0.000108221	0.003001656
ENSG00000271013	LRRC37A9P	-5.163794755	0.000111375	0.003071434
ENSG00000165935	SMCO2	4.411674387	0.000112538	0.003099073
ENSG00000234373	SNX18P7	-4.511471129	0.000114909	0.003150826
ENSG00000137959	IFI44L	-3.353101886	0.000115796	0.003170645
ENSG00000168765	GSTM4	-2.187069763	0.000119954	0.003275974
ENSG00000152154	TMEM178A	2.823111676	0.000120871	0.003290865
ENSG00000138092	CENPO	-2.366681985	0.000121395	0.003295816
ENSG00000126878	AIF1L	-4.213641152	0.000122027	0.003302547
ENSG00000120738	EGR1	3.159212904	0.000124835	0.003365468
ENSG00000205336	ADGRG1	-2.189536519	0.000125368	0.003370406
ENSG00000166106	ADAMTS15	-3.723045439	0.000128919	0.003446619
ENSG00000171914	TLN2	-2.812619899	0.000129175	0.003448666
ENSG00000073605	GSDMB	2.6351209	0.000129575	0.003449765
ENSG00000157399	ARSL	-2.243380078	0.000130303	0.003464379
ENSG00000168237	GLYCTK	-2.076157606	0.000132627	0.003516456
ENSG00000142623	PADI1	-3.07063776	0.000134188	0.003546498
ENSG00000127589	TUBBP1	-2.073675811	0.000134525	0.003547231
ENSG00000256393	RPL41P5	3.16132524	0.000135309	0.003563004
ENSG00000164050	PLXNB1	-2.141617954	0.000139555	0.003654827
ENSG00000196535	MYO18A	-2.126015482	0.00014218	0.003708441
ENSG00000281406	BLACAT1	-2.87422766	0.000143294	0.003727392

ENSG00000236778	INTS6-AS1	2.177402901	0.000145625	0.003772722
ENSG00000204482	LST1	4.045823004	0.000150328	0.003864527
ENSG00000059728	MXD1	3.028819664	0.000150373	0.003864527
ENSG00000231528	FAM225A	-3.397493736	0.000156024	0.003972627
ENSG00000231638	LUARIS	-4.835631205	0.000158827	0.004038652
ENSG00000145217	SLC26A1	-3.7665002	0.000161493	0.004090233
ENSG00000111816	FRK	-2.17191916	0.000164515	0.004141213
ENSG00000164949	GEM	2.213568297	0.000168617	0.004237221
ENSG00000228262	LINC01320	-3.623721648	0.000170111	0.004266474
ENSG00000178127	NDUFV2	2.780620881	0.000170225	0.004266474
ENSG00000141295	SCRN2	-2.096046556	0.000176532	0.004356484
ENSG00000145506	NKD2	3.482435286	0.000183662	0.004480758
ENSG00000231107	LINC01508	-2.319271248	0.000187299	0.004551957
ENSG00000074047	GLI2	-3.714830426	0.000188	0.004551957
ENSG00000234492	RPL34-DT	2.80969638	0.000188732	0.004558217
ENSG00000170214	ADRA1B	-2.188511959	0.000189427	0.004564075
ENSG00000251169	LINC01843	-3.045554861	0.000192598	0.004608071
ENSG00000197019	SERTAD1	2.321552875	0.000192665	0.004608071
ENSG00000214691	LINC01913	-5.979624049	0.000194637	0.004636848
ENSG00000100714	MTHFD1	-2.1144398	0.000198383	0.004697017
ENSG00000226674	TEX41	-3.93054564	0.00019983	0.004725465
ENSG00000010292	NCAPD2	-2.071235531	0.000202011	0.004759488
ENSG00000233929	MT1XP1	4.797260977	0.00020487	0.004803349
ENSG00000138411	HECW2	-3.071739552	0.00020628	0.00482026
ENSG00000253819	LINC01151	-4.590197278	0.000206343	0.00482026
ENSG00000070540	WIPI1	2.134452396	0.00020825	0.004835476
ENSG00000013016	EHD3	-2.760904435	0.000209554	0.00485403
ENSG00000117242	PINK1-AS	-2.064755865	0.000210735	0.004875532
ENSG00000114423	CBLB	2.041562587	0.000215655	0.004959429
ENSG00000152952	PLOD2	-2.165677691	0.000216838	0.004968942
ENSG00000174599	TRAM1L1	-2.065109712	0.000217879	0.004986867
ENSG00000113657	DPYSL3	-2.637762223	0.000220924	0.005047434
ENSG00000011332	DPF1	-4.738858609	0.000221253	0.005047434
ENSG00000160949	TONSL	-2.270938831	0.000230993	0.005194377
ENSG00000112667	DNPH1	-2.118052385	0.000243554	0.005419841
ENSG00000153822	KCNJ16	-4.604928321	0.000246599	0.005468637
ENSG00000196352	CD55	2.425192697	0.000247736	0.005487542
ENSG00000166589	CDH16	-2.56743808	0.000250561	0.005524682
ENSG00000142677	IL22RA1	-3.017115886	0.000251599	0.005541231
ENSG00000175711	B3GNTL1	-2.114689164	0.000254386	0.005596203
ENSG00000217801	LOC100288175	2.672321767	0.000265074	0.005776865
ENSG00000231187	LOC102724593	-4.177796544	0.000265299	0.005776865
ENSG00000183853	KIRREL1	-2.389855724	0.000266405	0.005787843
ENSG00000139998	RAB15	-2.846881357	0.000266773	0.005789309

ENSG00000285886	LOC124901671	5.146211121	0.000267074	0.005789309
ENSG00000234263	MAP3K5-AS1	3.092550015	0.000268738	0.005812284
ENSG00000227199	ST7-AS1	-2.87646272	0.00027179	0.005847476
ENSG00000143127	ITGA10	2.951670723	0.000273624	0.005871787
ENSG00000176771	NCKAP5	-4.053158215	0.000274795	0.005890343
ENSG00000196611	MMP1	5.141493148	0.000281303	0.00598321
ENSG00000116396	KCNC4	-2.16461914	0.00028202	0.005987513
ENSG00000106772	PRUNE2	-2.806205864	0.000282128	0.005987513
ENSG00000134183	GNAT2	3.090947386	0.000286862	0.006067924
ENSG00000111799	COL12A1	-2.539740349	0.000290264	0.0061197
ENSG00000213763	ACTBP2	-2.537716582	0.000296484	0.006216805
ENSG00000187867	PALM3	-5.57817314	0.000299681	0.006256576
ENSG00000241749	RPSAP52	3.526740501	0.000310103	0.006425366
ENSG00000163710	PCOLCE2	-2.307347111	0.000313687	0.006478718
ENSG00000277075	H2AC8	3.441536002	0.000314716	0.006493003
ENSG00000139597	N4BP2L1	2.321022723	0.000316127	0.006515132
ENSG00000091262	ABCC6	-2.820007808	0.000323357	0.006607527
ENSG00000158856	DMTN	-2.828684918	0.000324926	0.00661966
ENSG00000150625	GPM6A	-4.074777071	0.000328325	0.0066495
ENSG00000268041	ERFL	3.85670899	0.000336773	0.006766747
ENSG00000160781	PAQR6	2.81267028	0.000339163	0.006802003
ENSG00000029534	ANK1	-4.232290373	0.000341029	0.00682377
ENSG00000235387	SPAAR	-4.54749724	0.000342356	0.00683612
ENSG00000144810	COL8A1	-2.254673593	0.000343785	0.006857539
ENSG00000198520	ARMH1	2.69273972	0.000351164	0.006960634
ENSG00000162148	PPP1R32	2.602619763	0.000357376	0.007040426
ENSG00000228716	DHFR	-2.143763277	0.000357616	0.007040426
ENSG00000263528	IKBKE	-2.732283088	0.000368007	0.007185021
ENSG00000233230	LOC100506235	2.238611696	0.000377299	0.007321194
ENSG00000273062	RALGPS2-AS1	-4.473591548	0.000380212	0.007355473
ENSG00000227038	GTF2IP7	-2.424534756	0.000381765	0.007378108
ENSG00000270362	HMGN3-AS1	2.242590207	0.00038633	0.00744391
ENSG00000255277	ABCC6P2	-3.675685508	0.000388057	0.007469705
ENSG00000232759	STEAP1B-AS1	-2.435375696	0.000389629	0.007492474
ENSG00000104490	NCALD	-3.465918819	0.000393508	0.007559514
ENSG00000174370	KCNJ5-AS1	-2.024104748	0.000408939	0.007778327
ENSG00000180739	S1PR5	-2.91891237	0.000413056	0.00783342
ENSG00000131871	SELENOS	2.006765041	0.0004175	0.007882753
ENSG00000134569	LRP4	-2.099619146	0.000417705	0.007882753
ENSG00000171368	TPPP	2.790156988	0.000419858	0.007907875
ENSG00000218336	TENM3	-2.381829218	0.00042048	0.007911852
ENSG00000249328	LOC101927040	2.754393925	0.000423358	0.00794164
ENSG00000226279	RPL12P10	3.22429825	0.000431946	0.007992058
ENSG00000153253	SCN3A	-4.20446253	0.000432171	0.007992058

ENSG00000258586	LINC02274	-2.73859543	0.000435585	0.008015843
ENSG00000277224	H2BC7	3.587674706	0.000451878	0.008244746
ENSG00000257167	TMPO-AS1	-2.64603453	0.000452901	0.008247764
ENSG00000108852	MPP2	-2.220241002	0.000455915	0.00827915
ENSG00000188460	ACTBP11	-2.166559493	0.000464728	0.00837363
ENSG00000253910	PCDHGB2	-6.139556521	0.000477476	0.008534239
ENSG00000198590	APRG1	2.139530012	0.000477942	0.008534239
ENSG00000169087	HSPBAP1	2.083085884	0.000479601	0.008540096
ENSG00000168939	SPRY3	-2.3469322	0.000481721	0.008565315
ENSG00000224892	RPS4XP16	2.093558869	0.00048271	0.008571667
ENSG00000124564	SLC17A3	-3.179369226	0.000503524	0.008851447
ENSG00000230753	ZNF341-AS1	3.659206759	0.000513307	0.008998766
ENSG00000145362	ANK2	-2.111932853	0.000514364	0.009009089
ENSG00000115687	PASK	-2.204103063	0.000519244	0.009078052
ENSG00000006459	KDM7A	2.01015519	0.000521305	0.009105814
ENSG00000260219	CD2BP2-DT	-2.762149704	0.000523406	0.00913422
ENSG00000188985	DHFRP1	-2.332336022	0.000538273	0.009325348
ENSG00000161955	TNFSF13	-2.416914335	0.000542988	0.009373964
ENSG00000121577	POPDC2	2.229687772	0.000566861	0.009725044
ENSG00000255815	KRT8P11	6.559245503	0.000602901	0.010224888
ENSG00000253741	LNCOC1	-2.84215683	0.000604702	0.010233088
ENSG00000113140	SPARC	-2.396619061	0.000605511	0.010233088
ENSG00000241316	SUCLG2-DT	-2.100151774	0.000607173	0.010243187
ENSG00000143199	ADCY10	2.812288969	0.000614251	0.010326397
ENSG00000137491	SLCO2B1	-2.760307658	0.000617264	0.010367987
ENSG00000161618	ALDH16A1	-2.40308662	0.000625402	0.010468139
ENSG00000100628	ASB2	-3.572518968	0.000639052	0.010641102
ENSG00000163216	SPRR2D	6.868505535	0.000683084	0.011227619
ENSG00000134871	COL4A2	-2.066856444	0.000692932	0.011351706
ENSG00000038295	TLL1	-2.993806586	0.000695782	0.011388698
ENSG00000135406	PRPH	3.393905693	0.000711485	0.011606251
ENSG00000038945	MSR1	2.472815517	0.000715709	0.011645541
ENSG00000234719	NPIPB2	3.688082711	0.000718514	0.011668993
ENSG00000243902	ELFN2	-3.159368724	0.000724525	0.01174927
ENSG00000092969	TGFB2	-2.633436897	0.000739085	0.011965214
ENSG00000178033	CALHM5	-2.807348916	0.000747471	0.012041919
ENSG00000232303	DFFBP1	-2.017790265	0.00075341	0.012114375
ENSG00000030419	IKZF2	-2.15276014	0.000759379	0.012181066
ENSG00000134986	NREP	-2.293629334	0.00077145	0.012302933
ENSG00000237950	LINC02918	-2.589947533	0.000778321	0.012402237
ENSG00000165887	ANKRD2	-2.093461518	0.000798503	0.012650529
ENSG00000204173	LRRC37A5P	3.357571542	0.000809255	0.012789975
ENSG00000233237	LINC00472	-2.270241121	0.0008132	0.012789975
ENSG00000187185	LOC388282	-3.576586188	0.000821416	0.012865282

ENSG00000285737	LINC02680	3.553246403	0.000829136	0.012954572
ENSG00000115415	STAT1	-2.055477822	0.00083641	0.013025937
ENSG00000163638	ADAMTS9	-2.260121178	0.000848033	0.013122014
ENSG00000178803	ADORA2A-AS1	-4.163713689	0.000869219	0.013331954
ENSG00000227121	LINC02672	-4.436161981	0.000881946	0.013462784
ENSG00000119681	LTBP2	-2.192834951	0.000896572	0.01362124
ENSG00000188825	LINC00910	2.342676776	0.000902619	0.013680722
ENSG00000165300	SLITRK5	-3.305632604	0.000916316	0.013877396
ENSG00000130066	SAT1	2.356631155	0.000917367	0.013882389
ENSG00000141582	CBX4	2.02891235	0.000934743	0.014089946
ENSG00000181722	ZBTB20	-2.459096185	0.000937013	0.014105465
ENSG00000105696	TMEM59L	2.682805806	0.000947304	0.014195842
ENSG00000145632	PLK2	-2.305843041	0.000948138	0.014195842
ENSG00000251365	LINC02236	-3.430419315	0.000954797	0.014258248
ENSG00000251201	TMED7-TICAM2	2.693531529	0.000965554	0.014385409
ENSG00000164776	PHKG1	2.120892731	0.001003027	0.014811544
ENSG00000154678	PDE1C	-3.0169313	0.001003178	0.014811544
ENSG00000134627	PIWIL4	2.58343745	0.001012857	0.014915855
ENSG00000170049	KCNAB3	2.259370385	0.001026556	0.01503795
ENSG00000242193	CRYZL2P	-2.491167232	0.001052584	0.015303015
ENSG00000134668	SPOCD1	-2.694873684	0.001075175	0.015537565
ENSG00000112118	MCM3	-2.141921649	0.001103558	0.015876229
ENSG00000164675	IQUB	2.182082366	0.001148961	0.016377636
ENSG00000241570	PAQR9-AS1	-4.754913582	0.001150847	0.016378766
ENSG00000245904	BTG1-DT	2.304709092	0.001160527	0.016452562
ENSG00000171502	COL24A1	-4.028056115	0.001162423	0.016452562
ENSG00000145604	SKP2	-2.208248448	0.001176177	0.016537784
ENSG00000118004	COLEC11	3.951797536	0.001197406	0.016749098
ENSG00000243746	EEF1A1P10	-4.598587088	0.001198167	0.016749098
ENSG00000060762	MPC1	2.038245033	0.00120578	0.0168433
ENSG00000185862	EVI2B	2.975067824	0.001208957	0.016875432
ENSG00000179363	TMEM31	2.5856492	0.001220993	0.017031087
ENSG00000180279	LINC01869	4.190643771	0.00122206	0.017033625
ENSG00000080493	SLC4A4	-3.164895403	0.00122776	0.017075981
ENSG00000128242	GAL3ST1	-3.007584237	0.001257446	0.017326118
ENSG00000274386	TMEM269	2.464069538	0.001261337	0.017367289
ENSG00000091656	ZFHX4	-2.365794438	0.001267697	0.01742239
ENSG00000137834	SMAD6	-2.112562848	0.00128494	0.017616676
ENSG00000116254	CHD5	2.8317968	0.00130894	0.017818861
ENSG00000137501	SYTL2	-2.765210034	0.001310796	0.017818861
ENSG00000232415	ELN-AS1	2.285204524	0.001315047	0.017851442
ENSG00000219102	HNRNPA3P12	-2.90410823	0.001319853	0.01789144
ENSG00000187753	C9orf153	4.355102949	0.001336901	0.018035942
ENSG00000287200	LOC124902947	2.050212297	0.001337071	0.018035942

ENSG00000163606	CD200R1	2.944408031	0.001347951	0.018119206
ENSG00000134516	DOCK2	-2.749979301	0.001349608	0.018128826
ENSG00000198133	TMEM229B	-2.460816297	0.00136571	0.018306788
ENSG00000115884	SDC1	-2.072359204	0.001387431	0.018559175
ENSG00000276966	H4C5	2.863207927	0.001400727	0.018685091
ENSG00000115009	CCL20	2.824925233	0.001421446	0.018882961
ENSG00000189184	PCDH18	-2.933803243	0.001437656	0.019019544
ENSG00000189007	ADAT2	-2.354311696	0.001481095	0.019487076
ENSG00000112297	CRYBG1	-2.111055089	0.001488614	0.019572631
ENSG00000197599	CCDC154	2.844453548	0.001511453	0.019791807
ENSG00000132846	ZBED3	-2.532156581	0.001521848	0.01987385
ENSG00000178404	CEP295NL	-4.193957844	0.001525541	0.019908572
ENSG00000134539	KLRD1	2.391825417	0.001585912	0.020543216
ENSG00000173083	HPSE	-2.143445224	0.001589382	0.020574321
ENSG00000272146	ARF4-AS1	2.187401818	0.001596594	0.020612226
ENSG00000113368	LMNB1	-2.770964906	0.001600681	0.020623506
ENSG00000281103	TRG-AS1	-4.781643205	0.00160933	0.020721062
ENSG00000242337	INHCAP	2.065267508	0.001614264	0.020737007
ENSG00000203734	ECT2L	3.17503961	0.00161432	0.020737007
ENSG00000138079	SLC3A1	-2.887480823	0.001614877	0.020737007
ENSG00000014138	POLA2	-2.354173504	0.001619121	0.020769547
ENSG00000265763	ZNF488	-3.067731548	0.00161984	0.020769547
ENSG00000229525	DNPEP-AS1	3.032657595	0.001630673	0.020842509
ENSG00000104361	NIPAL2	-2.034940746	0.001641092	0.020947869
ENSG00000241935	HOGA1	-2.666171773	0.001647359	0.021000239
ENSG00000153714	LURAP1L	2.298866809	0.001685325	0.02125873
ENSG00000118997	DNAH7	-2.058861706	0.00173325	0.021650436
ENSG00000187193	MT1X	2.614364703	0.001745955	0.021766768
ENSG00000214391	TUBAP2	-2.070020338	0.001750867	0.021813879
ENSG00000139679	LPAR6	-2.832220072	0.001760224	0.021852844
ENSG00000164045	CDC25A	-2.053250679	0.001760806	0.021852844
ENSG00000106526	ACTR3C	-2.365483488	0.001790176	0.022092529
ENSG00000258708	SLC25A21-AS1	-2.270347633	0.001802657	0.022200471
ENSG00000182612	TSPAN10	-2.227848366	0.001817965	0.022346105
ENSG00000270276	H4C15	3.159880821	0.001822328	0.022364486
ENSG00000089692	LAG3	2.747415878	0.001822946	0.022364486
ENSG00000186577	SMIM29	2.126258676	0.00185471	0.022581477
ENSG00000166866	MYO1A	3.463245062	0.001875955	0.022768136
ENSG00000124496	TRERF1	-2.681124271	0.001930337	0.023302621
ENSG00000106714	CNTNAP3	-2.284778675	0.001946419	0.023445939
ENSG00000174567	GOLT1A	2.427216768	0.001958062	0.023532246
ENSG00000256340	ABCC6P1	-3.181012909	0.001958474	0.023532246
ENSG00000175728	LINC02873	-4.724411133	0.001993014	0.023813491
ENSG00000199691	RN7SKP173	2.053594689	0.002002391	0.023876955

ENSG00000136883	KIF12	-2.166695154	0.002010053	0.023942772
ENSG00000225472	NFIB-AS1	-2.28789599	0.002018448	0.023998208
ENSG00000164220	F2RL2	-3.105745921	0.0020208	0.024011345
ENSG00000230914	KIF19BP	3.542785762	0.002041169	0.024178741
ENSG00000188761	BCL2L15	-2.118836609	0.002063168	0.024301834
ENSG00000113578	FGF1	-4.286558134	0.002088824	0.024546872
ENSG00000262294	RPH3AL-AS2	-3.687287425	0.002093233	0.024579643
ENSG00000039600	SOX30	2.342858809	0.002118299	0.024768132
ENSG00000266235	MIR3176	-3.419245624	0.002128044	0.024795835
ENSG00000170091	NSG2	4.321812685	0.002166316	0.025105003
ENSG00000154556	SORBS2	-2.369115459	0.002205902	0.025425997
ENSG00000232801	SDCBPP3	2.034434778	0.00220835	0.025432113
ENSG00000158125	XDH	-2.410652892	0.00221621	0.025453356
ENSG00000165555	NOXRED1	2.060451206	0.002232834	0.025613722
ENSG00000104738	MCM4	-2.00665965	0.002248024	0.025741947
ENSG00000164037	SLC9B1	3.362018654	0.002249711	0.025745947
ENSG00000155849	ELMO1	-3.345821072	0.0022813	0.025960167
ENSG00000091622	PITPNM3	-2.164619994	0.0023371	0.026277364
ENSG00000079257	LXN	-2.183745363	0.002345368	0.026339553
ENSG00000234779	BNC2-AS1	-2.83988598	0.002368591	0.026483098
ENSG00000169903	TM4SF4	-2.376694666	0.002399552	0.026652607
ENSG00000181984	GOLGA8CP	-4.417425658	0.002440545	0.026896792
ENSG00000168398	BDKRB2	-2.431108019	0.002442663	0.026896792
ENSG00000170439	METTL7B	-2.478348631	0.002466404	0.027082612
ENSG00000103056	SMPD3	2.902487386	0.002471368	0.027106192
ENSG00000128590	DNAJB9	2.044693856	0.002554932	0.027769555
ENSG00000175287	PHYHD1	4.836908592	0.0025689	0.027889874
ENSG00000257135	ODC1-DT	2.0137661	0.00265193	0.028486047
ENSG00000267577	DNAAF3-AS1	2.842932565	0.002668057	0.028555052
ENSG00000270106	TSNAX-DISC1	3.974480356	0.002694305	0.028732789
ENSG00000138336	TET1	-2.831854953	0.002709304	0.0288448
ENSG00000186765	FSCN2	-3.337958856	0.002717605	0.028917178
ENSG00000153132	CLGN	2.309462119	0.002792226	0.029402382
ENSG00000230426	LINC01036	-2.091663419	0.002913963	0.030156379
ENSG00000237870	LOC102724434	-2.043435421	0.00294653	0.030409748
ENSG00000149633	KIAA1755	-3.691963128	0.002994161	0.0307057
ENSG00000143382	ADAMTSL4	-2.821310096	0.00302122	0.030917315
ENSG00000198821	CD247	3.486793941	0.003058172	0.031179425
ENSG00000258758	LOC440180	2.840203466	0.003077586	0.031294486
ENSG00000130176	CNN1	-2.985221199	0.003080188	0.031304405
ENSG00000002822	MAD1L1	2.831834336	0.003126141	0.031671096
ENSG00000236714	LINC01844	3.862780941	0.003261771	0.032803463
ENSG00000129596	CDO1	3.085418506	0.003304867	0.033096162
ENSG00000244414	CFHR1	-3.618779262	0.003332308	0.033221606

ENSG00000234678	ELF3-AS1	-2.114532714	0.003332329	0.033221606
ENSG00000232860	SMG7-AS1	2.667524425	0.003355448	0.03337934
ENSG00000253846	PCDHGA10	-2.425017773	0.003384754	0.033596629
ENSG00000102048	ASB9	-2.253667342	0.003430293	0.033894509
ENSG00000197822	OCLN	2.239919108	0.003431887	0.033894509
ENSG00000283599	LOC101059915	4.81349442	0.003435434	0.033912139
ENSG00000259370	LOC105370854	-2.306855499	0.003460553	0.034125091
ENSG00000208028	MIR616	2.044714641	0.003505348	0.034443314
ENSG00000099860	GADD45B	2.021931645	0.003540434	0.034713731
ENSG00000234183	H2AZ2-DT	3.921440923	0.003617183	0.035289995
ENSG00000239322	ATP6V1B1-AS1	-2.54571467	0.003624778	0.035346174
ENSG00000154133	ROBO4	2.356815982	0.003657247	0.035572692
ENSG00000270240	LOC105371745	4.309739826	0.003692095	0.035700958
ENSG00000162779	AXDND1	2.015079122	0.003702886	0.035759198
ENSG00000115232	ITGA4	-2.321914305	0.003731418	0.035902797
ENSG00000270923	TAS2R6P	2.619273681	0.00373346	0.035902797
ENSG00000186231	KLHL32	2.288426701	0.003747294	0.03600005
ENSG00000136531	SCN2A	-2.617195056	0.003789016	0.036249886
ENSG00000235448	LURAP1L-AS1	2.125432729	0.003884858	0.036891779
ENSG00000174059	CD34	-2.952823659	0.003951639	0.037347469
ENSG00000234362	LINC01914	-2.410255949	0.003980986	0.037538521
ENSG00000089127	OAS1	-2.252227725	0.004368461	0.040023091
ENSG00000271447	MMP28	-2.969654838	0.004371933	0.040035837
ENSG00000210127	TRNA	-2.015520785	0.004378712	0.040060305
ENSG00000106327	TFR2	-2.22278337	0.004414754	0.040312906
ENSG00000258469	CHMP4BP1	2.793902269	0.004624189	0.041750003
ENSG00000182759	MAFA	2.507415813	0.004906719	0.043485007
ENSG00000171557	FGG	2.399647446	0.005014855	0.044280252
ENSG00000169896	ITGAM	-2.005818226	0.005055335	0.044558021
ENSG00000170482	SLC23A1	-2.029039269	0.005067349	0.044582878
ENSG00000217275	RPS10P1	2.428008859	0.00518783	0.045144906
ENSG00000129675	ARHGEF6	-2.051070429	0.005200061	0.045230898
ENSG00000230641	USP12-DT	2.864109291	0.005290904	0.045731752
ENSG00000233452	STXBP5-AS1	2.064932708	0.005339836	0.046008774
ENSG00000170962	PDGFD	-2.100554342	0.005655066	0.047947738
ENSG00000286380	LOC105375501	2.640366714	0.005678976	0.04809266
ENSG00000110375	UPK2	2.01472005	0.005740361	0.048289152
ENSG00000232679	LINC01705	2.365348908	0.00596618	0.049501296
ENSG00000259343	TMC3-AS1	-2.071262945	0.006046868	0.04994489
ENSG00000240764	PCDHGC5	-2.073244172	0.006056362	0.04994489
ENSG00000142235	LMTK3	-2.176610985	0.006072722	0.049987776
ENSG00000091513	TF	2.189971425	0.006170564	0.050457596
ENSG00000242419	PCDHGC4	-2.617761194	0.006174064	0.050464778
ENSG00000047662	FAM184B	2.884846906	0.006295505	0.051045564

ENSG00000272666	KLHDC7B-DT	2.786452762	0.006319665	0.05119833
ENSG00000104368	PLAT	-2.005528337	0.006368162	0.051547835
ENSG00000256742	KDM2B-DT	-2.937963655	0.006687053	0.053211695
ENSG00000224509	MRPS9-AS2	2.48918301	0.006760022	0.053681382
ENSG00000233834	MACC1-DT	-2.132361346	0.007081837	0.055489321
ENSG00000223722	IFITM3P2	2.663272592	0.007093544	0.055510902
ENSG00000214814	FER1L6	2.176407622	0.007105269	0.055574693
ENSG00000057468	MSH4	2.572331667	0.007366845	0.057041194
ENSG00000168490	PHYHIP	-2.416810775	0.007552066	0.057893158
ENSG00000261083	LINC02516	-2.316223038	0.007593011	0.058022182
ENSG00000236591	UST-AS2	-2.546313583	0.007871621	0.05970088
ENSG00000251348	HSPD1P11	-2.467331328	0.007975843	0.060259465
ENSG00000204956	PCDHGA1	-2.66578074	0.007984466	0.060295401
ENSG00000210741	MIR196A1	-2.035353618	0.00840263	0.062157219
ENSG00000243417	RPS3AP18	2.314500298	0.009468834	
ENSG00000251000	GGCTP1	-2.197994174	0.010426961	0.07175852
ENSG00000255587	RAB44	2.156897684	0.010953597	0.074477493

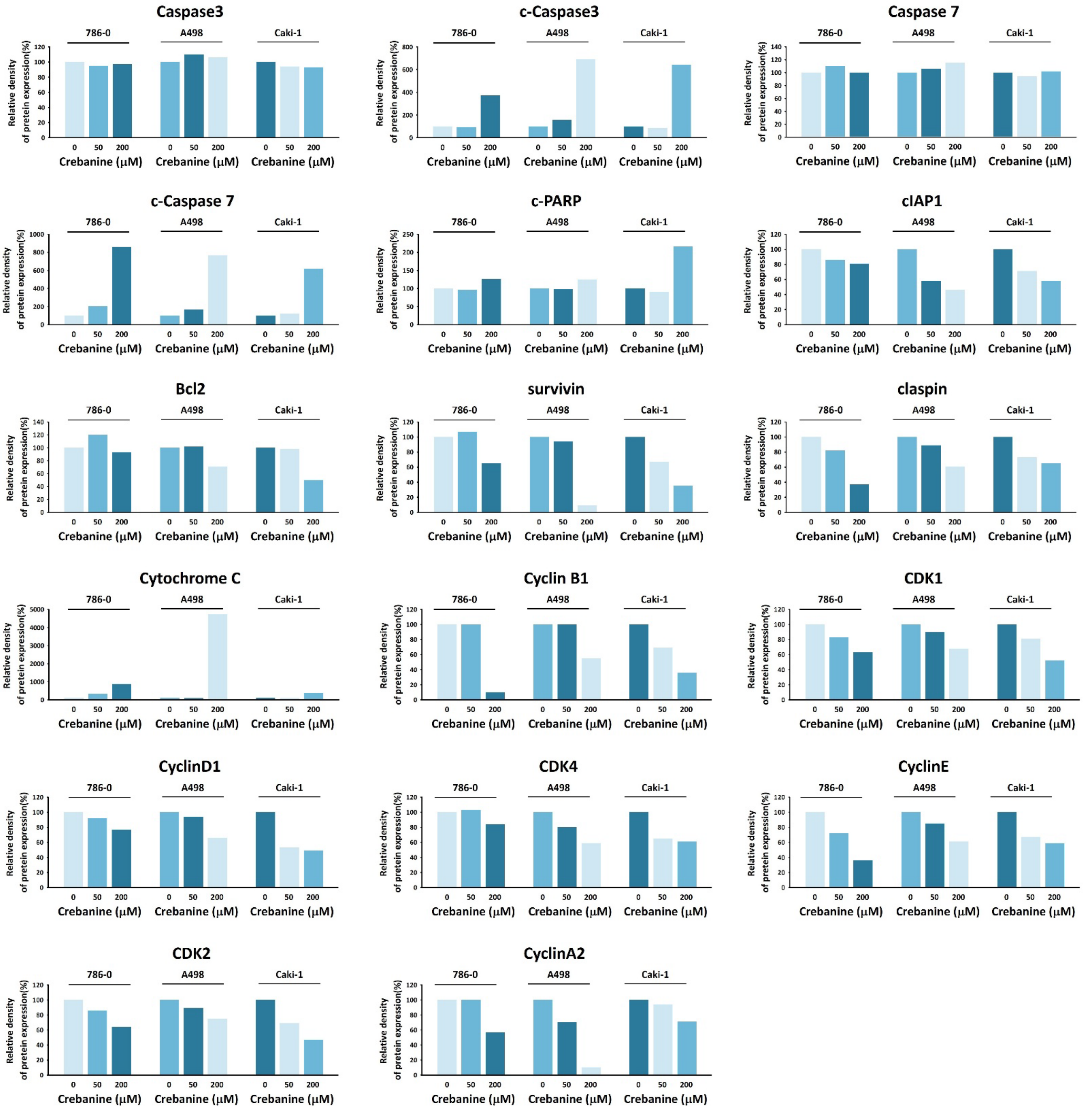

Supplementary Figure S2. Relative quantification of the indicated protein, presented in a bar graph.
